# Recurrent functional misinterpretation of RNA-seq data caused by sample-specific gene length bias

**DOI:** 10.1371/journal.pbio.3000481

**Published:** 2019-11-12

**Authors:** Shir Mandelboum, Zohar Manber, Orna Elroy-Stein, Ran Elkon

**Affiliations:** 1 School of Molecular Cell Biology and Biotechnology, George S. Wise Faculty of Life Sciences, Tel Aviv University, Tel Aviv, Israel; 2 Sagol School of Neuroscience, Tel Aviv University, Tel Aviv, Israel; 3 Department of Human Molecular Genetics and Biochemistry, Sackler School of Medicine, Tel Aviv University, Tel Aviv, Israel; Institute for Systems Biology, UNITED STATES

## Abstract

Data normalization is a critical step in RNA sequencing (RNA-seq) analysis, aiming to remove systematic effects from the data to ensure that technical biases have minimal impact on the results. Analyzing numerous RNA-seq datasets, we detected a prevalent sample-specific length effect that leads to a strong association between gene length and fold-change estimates between samples. This stochastic sample-specific effect is not corrected by common normalization methods, including reads per kilobase of transcript length per million reads (RPKM), Trimmed Mean of M values (TMM), relative log expression (RLE), and quantile and upper-quartile normalization. Importantly, we demonstrate that this bias causes recurrent false positive calls by gene-set enrichment analysis (GSEA) methods, thereby leading to frequent functional misinterpretation of the data. Gene sets characterized by markedly short genes (e.g., ribosomal protein genes) or long genes (e.g., extracellular matrix genes) are particularly prone to such false calls. This sample-specific length bias is effectively removed by the conditional quantile normalization (cqn) and EDASeq methods, which allow the integration of gene length as a sample-specific covariate. Consequently, using these normalization methods led to substantial reduction in GSEA false results while retaining true ones. In addition, we found that application of gene-set tests that take into account gene–gene correlations attenuates false positive rates caused by the length bias, but statistical power is reduced as well. Our results advocate the inspection and correction of sample-specific length biases as default steps in RNA-seq analysis pipelines and reiterate the need to account for intergene correlations when performing gene-set enrichment tests to lessen false interpretation of transcriptomic data.

## Abstract 2

Analysis of numerous RNA-seq datasets reveals a recurrent sample-specific length bias that causes frequent false positive calls by gene-set enrichment analyses, leading to functional misinterpretation of the data. Its removal requires methods that allow the integration of gene length as sample-specific covariate.

## Introduction

The ability to profile entire cellular transcriptomes, formerly by expression microarrays and subsequently by RNA sequencing (RNA-seq), has transformed biological research over the last two decades by turning the paradigm of systems-level analysis from a formidable task to one that is readily accessible to most experimental laboratories [1]. Consequently, RNA-seq is one of the most vastly used techniques in biological and biomedical research and is routinely applied for multiple goals, including the elucidation of key transcriptional networks driving different biological processes [2] and the identification of diagnostic and prognostic expression signatures for multiple diseases [3].

Data normalization is a critical component of RNA-seq processing pipelines, allowing for accurate estimation and detection of differential expression. The aim of normalization is to remove systematic effects that occur in the data to ensure that technical bias has minimal impact on the results [4–6]. Attesting the importance of this preprocessing step, numerous normalization methods have been developed for RNA-seq data over the last decade. Prominent among them are reads per kilobase of transcript length per million reads (RPKM) [7], edgeR's Trimmed Mean of M values (TMM) [8], DESeq's relative log expression (RLE) [9,10], and upper-quartile (UQ) normalization [11].

A well-known inherent technical effect in RNA-seq experiments relates to gene length and stems from the fact that in standard RNA-seq protocols, RNA (or cDNA) molecules are fragmented prior to sequencing in such a way that longer transcripts are sheared into more fragments than shorter ones are. Therefore, the number of reads for a given transcript is proportional not only to its expression level but also to its length. Thus, one of the most basic RNA-seq normalization methods, RPKM, divides gene counts by gene length (in addition to library size), aiming to adjust expression estimates for this length effect. A well-known consequence of the fact that longer genes tend to get more counts than equally expressed shorter genes is overrepresentation of long genes among the ones that pass statistical tests for differential expression (termed “length bias”), because of the increased statistical power [12,13].

Importantly, the way RPKM normalization handles the length effect is based on the assumption that this effect is the same for all samples. However, previous studies indicated that in addition to this universal length effect, gene length can affect expression measurement in a sample-specific manner. Importantly, removal of sample-specific technical effects requires normalization methods that allow for correction of sample-specific covariates. Two such methods are conditional quantile normalization (cqn) [14] and EDASeq [15]. cqn combines generalized regression to remove sample-specific biases and quantile normalization to equalize the shape and scale of gene-expression distribution across samples [14]. EDASeq implements two normalization steps: a within-sample normalization step that adjusts for gene-specific and sample-specific effects and a between-sample normalization that corrects distributional differences between samples [15]. Although both studies emphasized the effect of sample-specific GC-content biases, the packages implementing these methods provide correction for both sample-specific GC and length effects.

Gene-set enrichment analyses (GSEAs) are among the most vastly used techniques for functional interpretation of gene-expression data, and numerous statistical methods were developed over the last two decades for this task [16–19]. Notably, in addition to technical biases, flaws in statistical tests for gene-set enrichment were also shown as a main cause for functional misinterpretation of transcriptomic data [20,21]. Specifically, many methods for GSEA assume that individual genes are independent. However, this assumption is clearly violated, as many gene sets contain co-regulated genes. Importantly, it was shown that methods based on the independence assumption produce very high false positive rates and that gene sets with high intergene correlation are especially susceptible to false calls [20,22,23]. Therefore, statistical methods that account for intergene correlation within gene sets were developed in recent years [23,24].

In this study, analyzing numerous publicly available RNA-seq datasets, we found that sample-specific length effects have greater impact on expression measurements than currently appreciated. If left uncorrected, sample-specific length effects make the comparison of expression level of a gene between samples problematic and distort fold-change (FC) estimates. We found that the coupling between FC estimates and genes' length, caused by sample-specific length effects, recurrently prompt false results by gene-set enrichment tests that assume gene independence. In addition, we observed that gene sets characterized by exceptionally short genes (e.g., ribosomal protein genes) or long genes (e.g., extracellular matrix [ECM] genes) are particularly prone to such false calls. Allowing for the integration of gene length as sample-specific covariate, cqn and EDASeq effectively remove this length bias and thus substantially reduce false results while retaining true ones. Notably, false calls were also attenuated when we applied gene-set methods that account for intergene correlation.

## Results

### A prevalent sample-specific technical effect in RNA-seq data links differential expression to gene length

**Fig 1:**
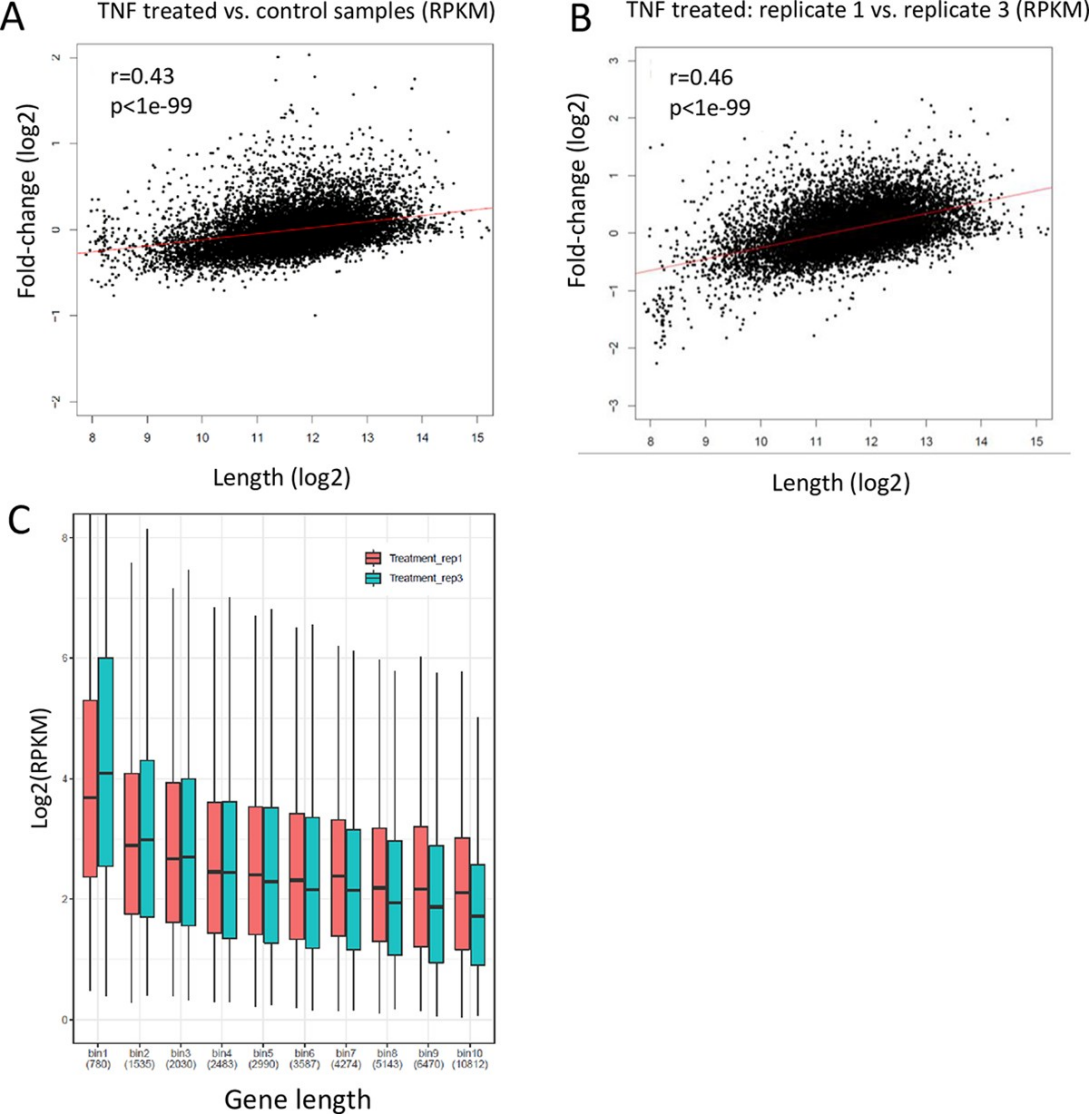
Sample-specific length effect couples differential gene expression and length in RNA-seq data. (A) An RNA-seq experiment that measured gene-expression profiles in TNF and vehicle-treated samples (both silenced for REL-A) (GEO accession: GSE64233) shows a significant coupling between gene length and FC of expression levels (after RPKM normalization of gene counts and averaging over three replicate samples of each condition). Spearman's correlation coefficient is indicated together with its statistical significance. The red line is the linear regression line. (See S1 Fig and S1 Table for results on a collection of 35 publicly available RNA-seq datasets.) Note that throughout the paper, gene length refers to the length of the gene's principal transcript (Methods). (B) Same analysis as in (A), but here the comparison is between two individual replicate samples of the same biological condition (TNF-treated cells silenced for REL-A replicate 1 versus replicate 3, as defined in S2 Table). (By definition, differences in gene expression between replicates reflect experimental technical effects.) Note that in both (A) and (B), data were RPKM-normalized before FC calculation, supposedly accounting for the length effect. Still, there is a technical coupling between FC and length. (C) Sample-specific length effect. Analyzing the two replicate samples from (B), we split the genes into 10 equally sized bins according to length (approximately 1,210 genes in each bin) and examined the distribution of gene expression in each bin. The length effect on expression markedly varies between these two replicates: shorter genes (bins 1–3) show higher expression in replicate 3, whereas longer genes (bins 8–10) show elevated expression in replicate 1. This sample-specific length bias underlies the strong technical link between differential expression and gene length that is shown in (B). (Average length in each bin is indicated below the bins.) Data underlying the results presented in this figure are provided in S1 Data. FC, fold change; GEO, Gene Expression Omnibus; RNA-seq, RNA sequencing; RPKM, reads per kilobase of transcript length per million reads; TNF, tumor necrosis factor.

Analyzing numerous publicly available RNA-seq datasets, we frequently observed a coupling between gene-expression FC and gene length (Fig 1A). Collectively, we analyzed 35 human and mouse RNA-seq datasets selected from recent Gene Expression Omnibus (GEO) studies (mostly published within the last 2 years) covering together a diverse spectrum of biological conditions. We detected a strong statistical relationship (*p* < 10^−8^) between gene length and FC in treated versus control samples, across the vast majority of the datasets (85%; 30 out of the 35 datasets). The magnitude of the coupling varied considerably over these 30 datasets, with some showing very strong bias, whereas others showed only a subtle one. The median Spearman's correlation between gene length and (log) FC was 0.18 (range: 0.05–0.43) (S1 Fig). To rule out the possibility that this recurrent relationship resulted from any particular way that we normalized the data, we analyzed each dataset by five of the most widely used RNA-seq normalization methods: RPKM [7], TMM [8], quantile normalization [25], RLE [9], and UQ normalization [11] (see Methods). Importantly, the coupling persisted regardless of processing method (S1 Table). We also analyzed the original gene-level summaries as produced by the authors of these 35 datasets (obtained from GEO) and found similar results, further precluding the possibility that the unexpected link we observed between gene length and FC is caused by any specific data-processing pipeline or any flaw in the analysis.

Puzzled by the prevalent link between gene length and FC, we next asked whether it reflects a genuine biological effect or rather stems from some experimental artifact. To address this question, we examined FC estimates between replicate samples within each dataset. By definition, differences in gene expression between replicates reflect experimental technical effects (that is, these differences are not due to the biological factors of interest). Notably, virtually all 35 datasets showed a significant (*p* < 10^−8^) relationship between gene length and FC in comparisons between replicate samples (Fig 1B and S2 Fig). Considering all pairwise comparisons between replicate samples and taking from each dataset the pair showing the strongest length–FC coupling, we found that the median length–FC Spearman's correlation was 0.22 (range: 0.05–0.67) over all 35 datasets (S2A Table and S2B Table).

**Fig 2:**
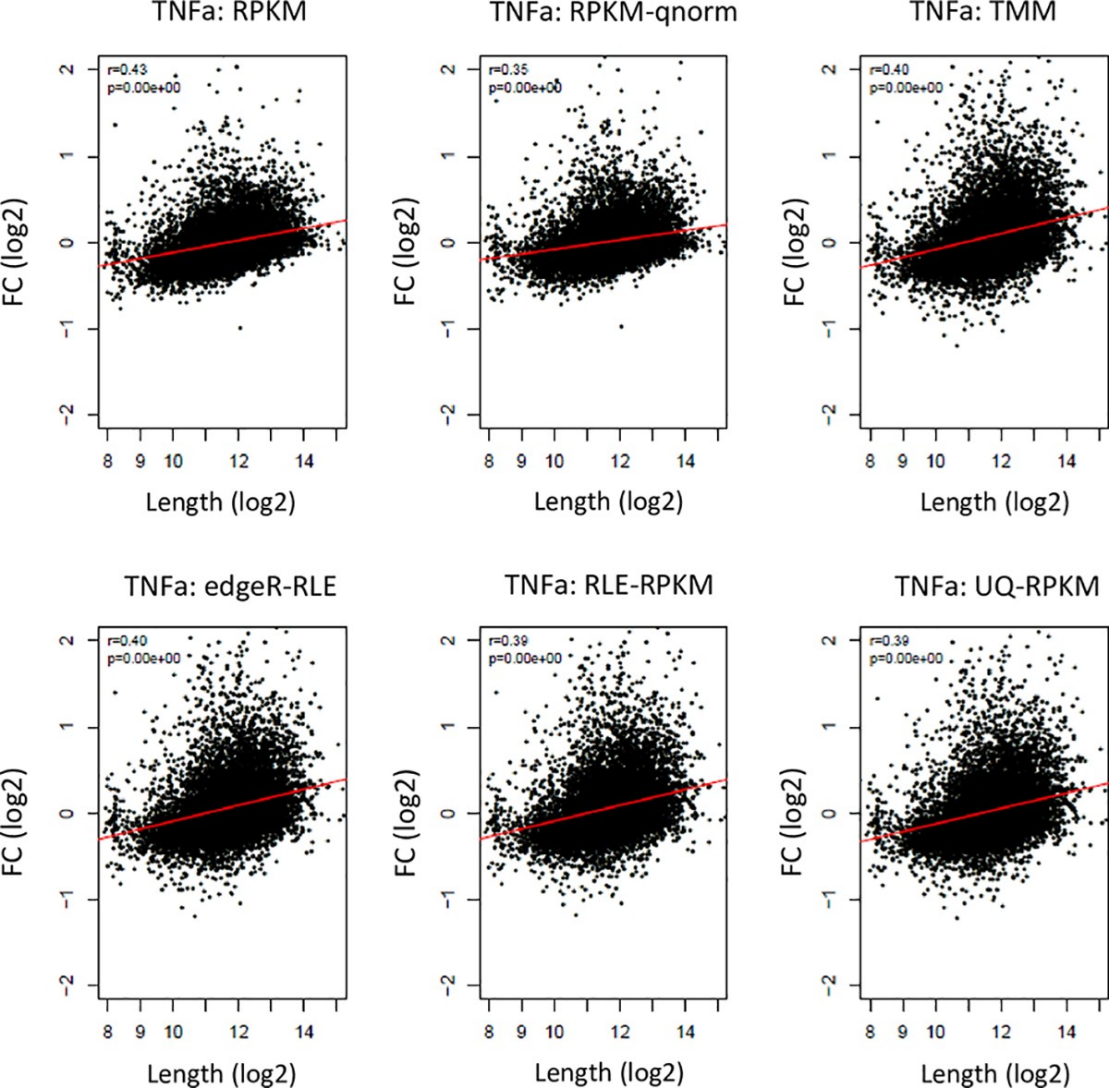
Sample-specific length bias is not removed by widely used RNA-seq normalization methods. We applied to the RNA-seq data shown in Fig 1B, comparing two replicate samples, six of the most popular normalization methods (RPKM, RPKM followed by qnorm, TMM normalization with FC estimation using edgeR model fit, RLE normalization with FC estimation using edgeR model fit, RLE followed by RPKM, and UQ followed by RPKM). Importantly, none of these methods removed the technical coupling between FC and gene length in this technical comparison. Data underlying the results presented in this figure are provided in S1 Data and in https://github.com/ElkonLab/RNA-seq_length_bias. FC, fold change; qnorm, quantile normalization; RLE, relative log expression; RNA-seq, RNA sequencing; RPKM, reads per kilobase of transcript length per million reads; TMM, Trimmed Mean of M values; TNFa, tumor necrosis factor alpha; UQ, upper quartile.

Collectively, our observations indicate that the effect of gene length on RNA-seq expression measurements varies between different samples because of some stochastic technical effects and that such sample-specific bias leads to coupling between expression FC and gene length (Fig 1C). Hereafter, we refer to this association as “sample-specific length bias” (to distinguish it from the well-documented “length bias” in RNA-seq data, which we discussed in the Introduction). Notably, none of the five alternative RNA-seq normalization methods that we applied (RPKM, TMM, quantile normalization, RLE, and UQ normalization) removed the sample-specific length bias from these technical comparisons (Fig 2; S2A Table and S2B Table—S2A Table contains results for the pairs showing the strongest length effect in each dataset, and S2B Table shows the results for all pairwise comparisons).

### Sample-specific length bias leads to false positive calls by GSEA

**Fig 3:**
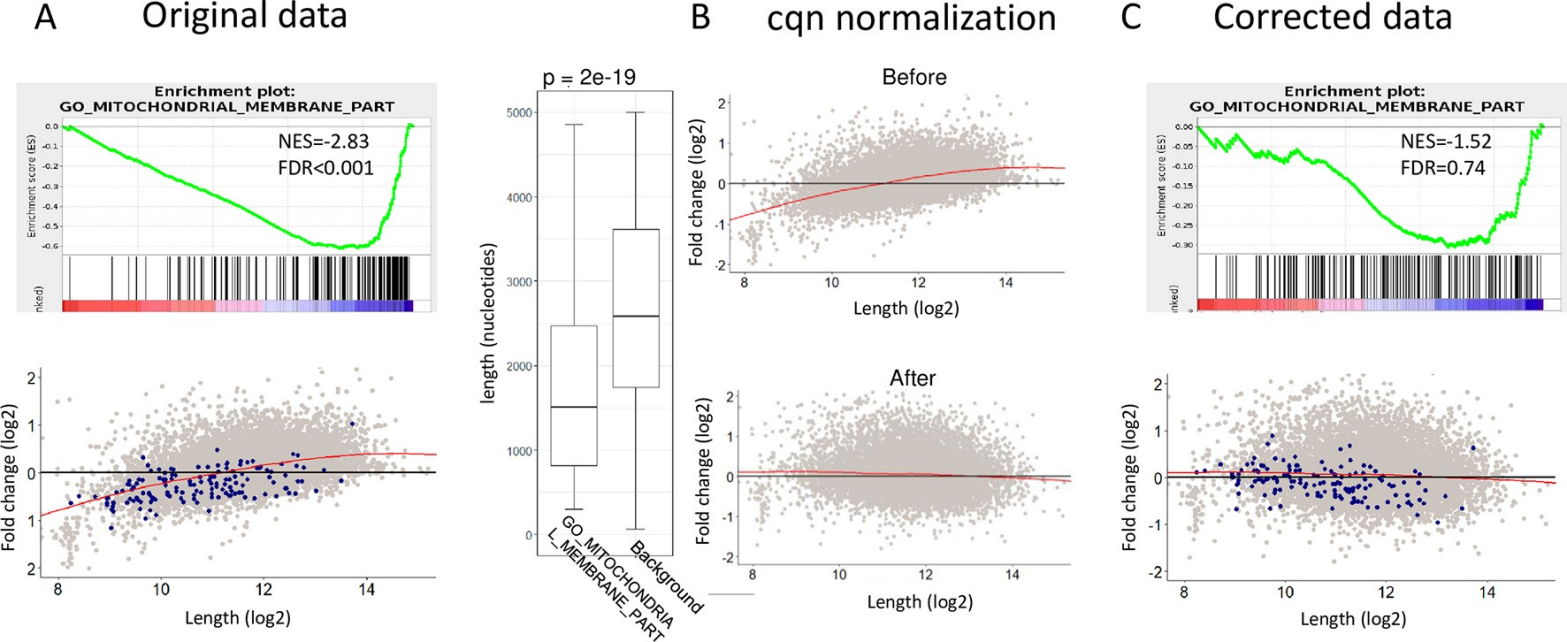
Sample-specific length bias leads to false positive results by GSEA. (A) As an example, GSEA analysis applied to the comparison between the two replicate samples shown in Fig 1B detects the GO category "mitochondrial-membrane-part" as a significantly enriched gene set (FDR < 0.001) (top). Genes assigned to the "mitochondrial-membrane-part" category are colored in blue in the scatter plot (red line is a lowess line) (bottom). Genes assigned to this GO category are significantly shorter than the set of all other genes expressed in the dataset (background set shown in gray) (*p*-value calculated using Wilcoxon test) (right). (B) cqn was applied to correct sample-specific length effects and cancel the coupling between gene length and differential expression. (C) Same GSEA analysis as in (A) but performed here after the data were corrected by cqn. Notably, cqn canceled the sample-specific length bias, and consequently, the GO category mitochondrial-membrane-part is no longer enriched. (See S3 Fig for numerous additional examples.) Data underlying the results presented in this figure are provided in S2 Data. cqn, conditional quantile normalization; FDR, false discovery rate; GO, Gene Ontology; GSEA, gene-set enrichment analysis; lowess, locally weighted scatterplot smoothing; NES, normalized enrichment score.

Functional interpretation of RNA-seq data is usually based on initial detection of sets of differentially expressed genes (DEGs), followed by their functional characterization, commonly through identification of functional categories (e.g., Gene Ontology categories) that the DEG sets are enriched for [26]. However, RNA-seq experiments often include only a small number of replicate samples (mostly 1–3 replicates per condition), which limits the statistical power of tests for DEG detection. An attractive alternative statistical approach for functional interpretation of transcriptomic data is provided by the framework of GSEAs [19]. Instead of focusing on the set of DEGs, GSEA considers all the genes expressed in a dataset and ranks them based on a score of differential expression between the compared samples (e.g., FC or T score calculated between treated and control samples). The ranked gene list is then tested against a large number of curated gene sets, seeking those whose genes are significantly concentrated at either end of the expression list (each end represents, respectively, induced and repressed genes). This powerful method builds on the amplification of weak signals, achieved by considering the coordinated response of many genes that function in the same process, in which individually most of them show only mild change in expression that does not reach statistical significance in per-gene tests. However, this increased sensitivity makes GSEA tests especially susceptible to false positive calls that stem from mild experimental artifacts. As gene length is also associated with biological function (e.g., ECM genes, like collagens and integrins, are notably long, whereas housekeeping genes are markedly short [27]), we suspected that the technical coupling that we observed between gene length and differential expression would result in GSEA false findings. To examine the impact of sample-specific length biases on GSEA results, we ran this analysis on comparisons between replicate samples (in which, by definition, all calls are false positives, in the sense that they stem from technical effects rather than biological factors of interest). Performing these technical tests on multiple RNA-seq datasets, we found that GSEA regularly detected hundreds of enriched gene sets (also after correcting for multiple testing), many of which were accounted for by the sample-specific length bias. Consequently, gene sets detected by GSEA in such technical comparisons between replicate samples were frequently characterized by markedly long or short genes (Fig 3A and S3 Fig). These results demonstrate that sample-specific length effects recurrently cause GSEA false positive calls, leading to functional misinterpretation of RNA-seq data.

### Correction of sample-specific length effects reduces false positive calls by GSEA

Removal of sample-specific technical effects requires normalization methods that allow for correction of sample-specific covariates. We therefore next applied cqn [14] and EDASeq [15] normalization to the datasets we analyzed. As these two studies mainly focused on sample-specific GC-content biases, we first examined GC biases in the 35 RNA-seq datasets. In this collection of datasets, we observed that GC and length biases showed similar magnitude (over the 35 datasets, the median [mean] absolute Spearman's correlation between gene length and FC and between gene GC content and FC was 0.16 [0.15] and 0.17 [0.17], respectively; S1 Table and S3 Table). Next, as there is some general relationship between genes’ GC content and length, we examined whether removal of the GC effect also corrects for the length bias. We found that in most datasets this was not the case and that effective removal of the length effect required using sample-specific gene-length covariate (S4A Fig and S4B Fig; S4 Table). Overall, running cqn with only GC content as sample-specific covariate failed to correct the length effect in 25 out of the 30 datasets that showed significant length bias. In contrast, including the sample-specific gene-length covariate effectively attenuated the length bias in all the 30 datasets and completely removed it (*r* < 0.05 after normalization) in 26 of them (S4 Table). Running EDASeq with a sample-specific length covariate also effectively corrected the length effect in all datasets (S4 Table). In subsequent analyses, we continued using cqn. Importantly, as cqn successfully removed the technical length effect, it consequently markedly reduced GSEA false positive results that were called in comparisons between replicate samples (Fig 3B and 3C and S3 Fig).

**Fig 4:**
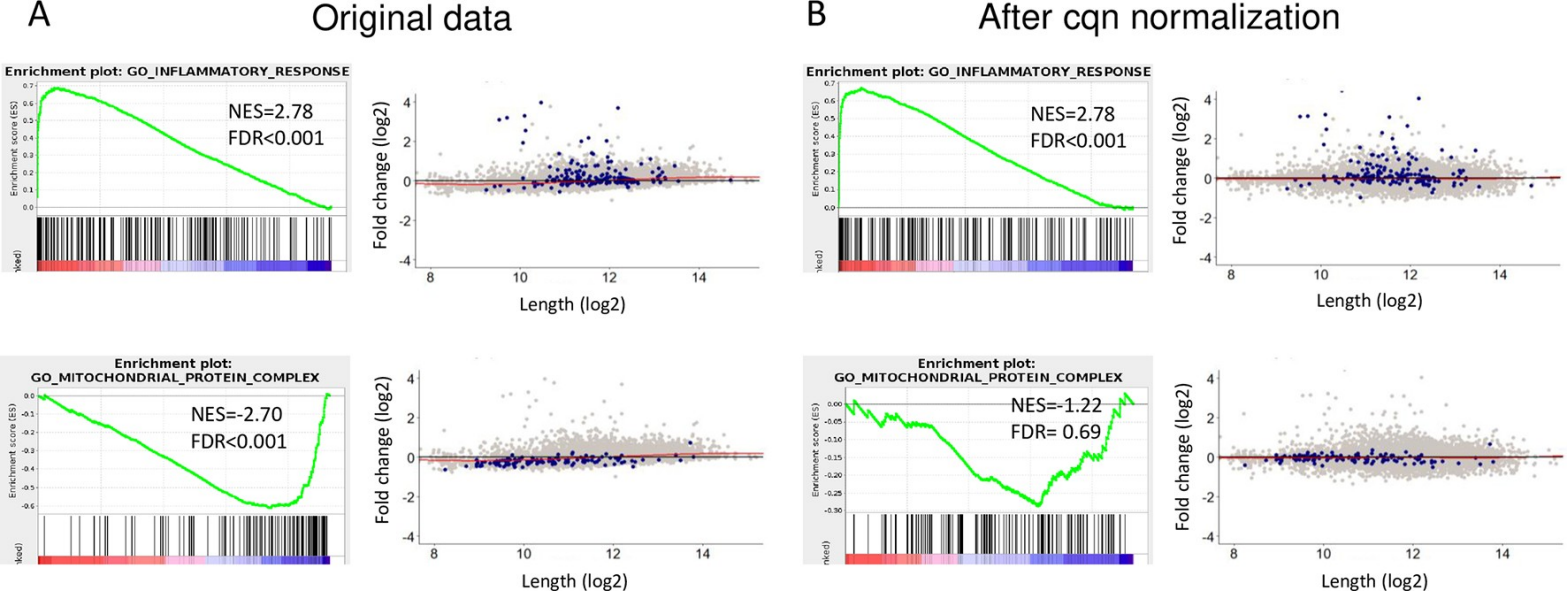
Sample-specific length bias correction by cqn reduces GSEA false calls without compromising the detection of true ones. (A) Application of GSEA to the original data comparing TNF- and vehicle-treated samples (Fig 1A) detects both biologically true gene sets (in this example, the GO category “inflammatory response”) and false gene sets that stem from the FC–length technical effect (in this example, the GO category “mitochondrial protein complex”). (B) After cqn, the false call is no longer significant, and the detection of the genuine set is not compromised. (See S5 Fig for additional examples.) Data underlying the results presented in this figure are provided in S2 Data. cqn, conditional quantile normalization; FC, fold change; FDR, false discovery rate; GO, Gene Ontology; GSEA, gene-set enrichment analysis; NES, normalized enrichment score; TNF, tumor necrosis factor.

Demonstrating that cqn alleviates GSEA false positive calls that originate from sample-specific length biases, we next confirmed, using multiple RNA-seq datasets, that cqn correction does not compromise GSEA detection of true biological responses. For example, in the dataset that examined transcriptional responses to tumor necrosis factor (TNF) treatment, although cqn canceled the false detection of “mitochondrial protein complex” (containing markedly short genes), it did not compromise the call of the true gene-set “inflammatory response” (Fig 4). We confirmed this utility of cqn on additional datasets (S5 Fig).

**Fig 5:**
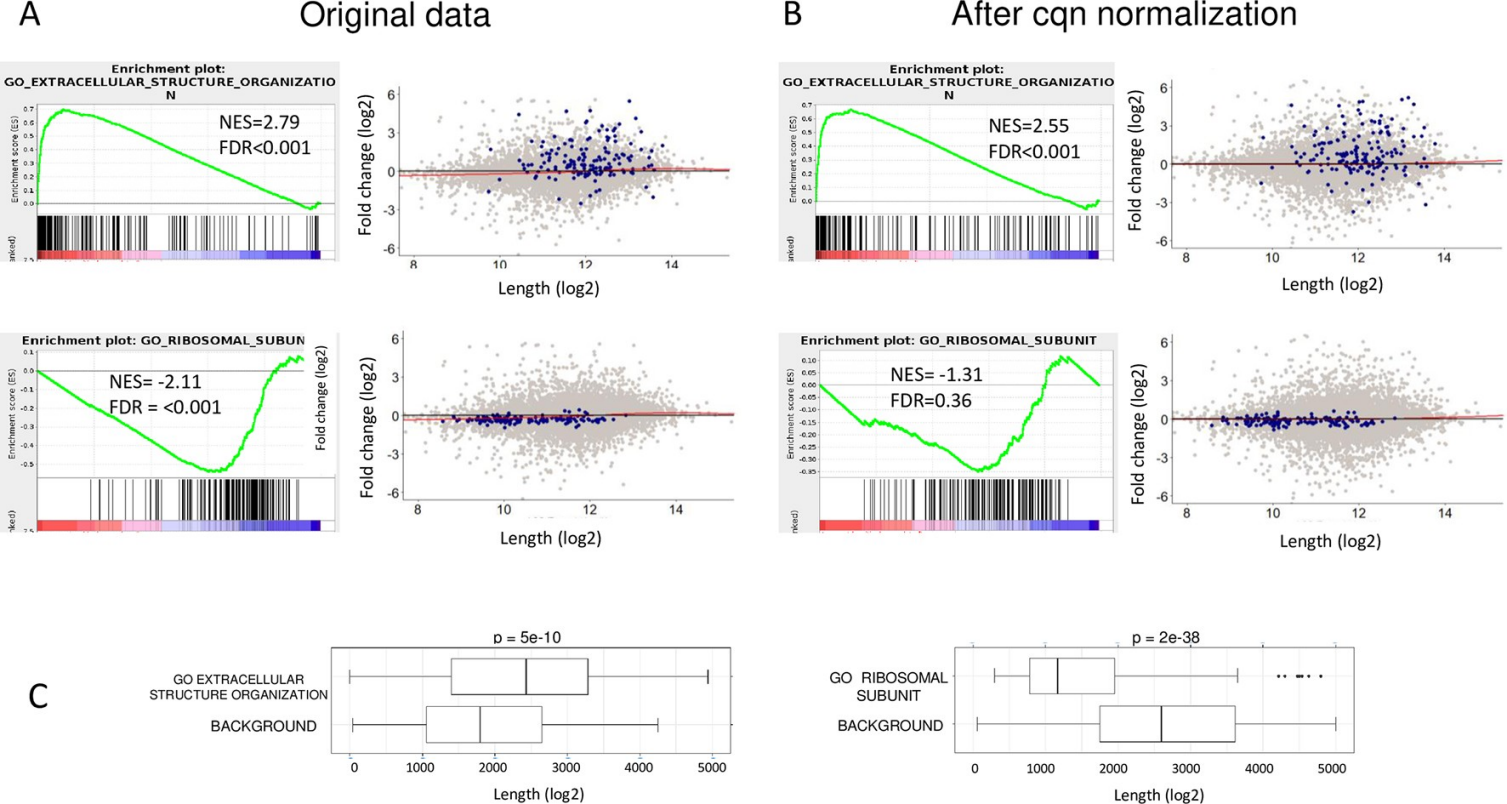
cqn correction in an EMT dataset as a test case in which the true biological response is genuinely coupled to gene length. EMT is known to involve strong induction of ECM genes. (A) True and false gene sets (GO “extracellular structure organization” and GO “ribosomal subunit” gene sets, respectively) detected by GSEA on RPKM-normalized EMT RNA-seq data (GSE114572). (B) cqn correction does not compromise the detection of the true set (ECM) but abolishes the false one (ribosomal subunit). (C) Length distribution of genes assigned to the GO extracellular structure organization and GO ribosomal subunit gene sets. Data underlying the results presented in this figure are provided in S3 Data. cqn, conditional quantile normalization; ECM, extracellular matrix; EMT, epithelial–mesenchymal transition; FDR, false discovery rate; GO, Gene Ontology; GSEA, gene-set enrichment analysis; NES, normalized enrichment score; RNA-seq, RNA sequencing; RPKM, reads per kilobase of transcript length per million reads.

Last, we sought to examine the effect of cqn correction in a more challenging test case, in which the true biological response is genuinely coupled to gene length. For this task, we analyzed an RNA-seq dataset that recorded gene-expression profiles in the transition of cells from epithelial to mesenchymal states (EMT). This physiological transition is known to involve drastic changes in the expression of the markedly long genes that encode ECM proteins (e.g., collagens and integrins). Importantly, although in this test case too did cqn remove false positive calls caused by sample-specific length effects, it did not compromise at all the detection of true ECM-related gene sets (Fig 5).

### Accounting for intergene correlation reduces false positive calls caused by sample-specific length effects

The statistical tests applied by the GSEA analyses that we carried out are based on the assumption that genes are independent. However, this assumption is clearly false in gene-expression datasets, as many genes are transcriptionally co-regulated and thus show highly correlated expression. Previous studies showed that such intergene correlation produces a substantial amount of variance inflation in the test statistic, which in turn causes a high rate of false positive results in GSEAs [20,21,28]. Consequently, statistical methods that account for intergene correlation in gene-set tests were developed in recent years, including Correlation Adjusted MEan RAnk gene-set test (CAMERA) [24], implemented in the limma package. We next sought to examine whether adjustment for intergene correlation could alleviate false calls that stem from the gene length–FC bias. We therefore applied CAMERA to seven RNA-seq datasets, presented in Fig 5 and S5 Fig, on which the independence assumption–based GSEA procedure detected both true and false gene-set enrichments. Interestingly, in all these seven datasets, CAMERA eliminated the false gene-set call (Table 1). However, CAMERA also showed reduced sensitivity, as it missed the true gene set in two datasets and because in the other five, although the true gene sets showed nominal statistical significance (*p* < 0.05), they did not pass significance threshold after correcting for multiple testing (Table 1). The reduction of false calls by CAMERA on these datasets suggests that, at least in part, the sample-specific gene length effect that we recurrently observed in RNA-seq datasets is related to markedly strong co-regulation (and thus exceptionally highly correlated expression) manifested by sets of genes featuring very short or long genes (see Discussion).

**Table 1:** 

Dataset	Gene set	True/False call	*p*-Value* No adjustment for intergene correlation	*p*-Value* Applying adjustment for intergene correlation
GSE64233	GO_INFLAMMATORY_RESPONSE	TRUE	2.02 × 10^−24^	0.007
	GO_MITOCHONDRIAL_PROTEIN_COMPLEX	FALSE	2.82 × 10^−18^	0.19
GSE76771	GO_RESPONSE_TO_ENDOPLASMIC_RETICULUM_STRESS	TRUE	2.83 × 10^−22^	0.002
	GO_INNER_MITOCHONDRIAL_MEMBRANE_PROTEIN_COMPLEX	FALSE	1.08 × 10^−23^	0.23
GSE101738	KEGG_P53_SIGNALING_PATHWAY	TRUE	1.50 × 10^−9^	0.011
	GO_ELECTRON_TRANSPORT_CHAIN	FALSE	1.81 × 10^−6^	0.46
GSE106847	GO_ENDOPLASMIC_RETICULUM_LUMEN	TRUE	2.70 × 10^−27^	0.003
	GO_INNER_MITOCHONDRIAL_MEMBRANE_PROTEIN_COMPLEX	FALSE	1.88 × 10^−10^	0.39
GSE84989	GO_RESPONSE_TO_ENDOPLASMIC_RETICULUM_STRESS	TRUE	6.72 × 10^−13^	0.175
	KEGG_RIBOSOME	FALSE	4.14 × 10^−7^	0.59
GSE42509	GO_SISTER_CHROMATID_SEGREGATION	TRUE	2.53 × 10^−16^	0.076
	GO_PROTEIN_LOCALIZATION_TO_ENDOPLASMIC_RETICULUM	FALSE	2.58 × 10^−9^	0.47
GSE114572	GO EXTRACELLULAR STRUCTURE ORGANIZATION	TRUE	8.87 × 10^−25^	0.048
	GO RIBOSOMAL SUBUNIT	FALSE	2.44 × 10^−18^	0.137

*The *p*-values reported here are without correction for multiple testing.

## Discussion

In this study, we report on a highly prevalent technical bias in RNA-seq datasets that is related to gene length and affects the functional interpretation of results obtained by this technology. This bias is not corrected for by many widely used RNA-seq normalization methods, as its removal requires the consideration of gene length as a sample-specific covariate. We show the effectiveness of cqn and EDASeq in correcting for this bias and demonstrate that their application markedly reduces GSEA false positive calls while retaining true results.

The original cqn [14] and EDASeq [15] publications emphasized sample-specific biases related to GC content. Sample-specific effects related to gene length are largely overlooked by current transcriptomic studies. The collection of 35 datasets analyzed in our study show that the impact of sample-specific length biases is much higher than currently appreciated, and in this dataset ensemble, it has a comparable magnitude to the effect related to GC content (S3 Table). As the sample-specific length effect detected by our study seems to randomly affect different samples (as evident by its rather stochastic behavior between replicate samples of the same biological condition), averaging over replicates is expected to attenuate its magnitude, and thus, datasets with a lower number of replicate samples are more likely to suffer from this technical issue. All 35 datasets analyzed in our study have 2–4 replicates, which is the typical size in small-scale RNA-seq experiments. Our results show that for studies of this scale, the length bias poses a considerable concern that should be accounted for to lessen false interpretation of the data.

Unexpectedly, the results we obtained using CAMERA (Table 1) suggest that the sample-specific length bias is, at least in part, related to the issue of intergene correlation. Importantly, previous studies demonstrated that gene-set testing procedures that are based on the statistical assumption that genes are independent are highly sensitive to intergene correlation. Gatti and colleagues demonstrated that gene sets with high internal gene correlation are especially prone to false calls [24] and that gene sets related to translation/ribosomal complexes tend to show particularly high levels of internal correlation (personal communication, D. Gatti to R. Elkon). Remarkably, these gene sets (translation/ribosomal complexes) feature markedly short genes and were among the false sets most frequently called by our GSEA analyses (S3 Fig and Fig 5). Of note, in our analyses, we used the pre-ranked GSEA method, which applies gene permutation for generation of the null distributions. Gene permutation breaks the structure of intergene correlations within a gene set and, in fact, reflects the unrealistic assumption that genes are independent. This makes pre-ranked GSEA highly prone to false positive results. On the other hand, the original GSEA method [23] permutes samples (rather than genes), thereby preserving intergene correlations within each gene set [20,29]. Therefore, this method is likely less sensitive to the length bias observed by our study. However, sample permutation is only effective for datasets with a large number of replicate samples, whereas small datasets (like the ones analyzed in our study, mostly probing two biological conditions, each with 2–4 replicates) have too few samples to support their robust permutation. CAMERA [24] offers a statistical method that accounts for inert-gene correlation in gene-set tests and is also applicable for small datasets, but our results suggest that it may have reduced power.

We still do not understand the exact factors that cause the sample-specific length effect. In our analyses, the correlation between gene length and FC was recurrently highly significant (S1 Table and S2 Table). Simulation shows that for transcriptome-scale analyses, even a mild correlation (approximately 0.1) between the shortest (or longest) genes in a dataset, on the background of no correlation between all the other genes, still frequently results in highly significant overall length–FC correlation (in 663 out of 1,000 such random simulations, we obtained length–FC correlation *p*-value below 10^−5^). This observation lends support to the hypothesis that sets with very short genes (e.g., ribosomal protein genes) or very long genes (e.g., ECM genes) also feature exceptionally tight co-regulation that is not related to any specific biological condition and that this (incidental) coupling between gene length and extent of gene–gene correlation contributes to the gene length–FC link that we recurrently observed in RNA-seq datasets.

Taken together, our study reports on a prevalent sample-specific length effect in RNA-seq data. We therefore recommend inspection for this bias and the usage of normalization methods that support gene-level sample-specific covariates as default steps in RNA-seq data analysis pipelines. In addition, our results reiterate the need to account for intergene correlations when performing gene-set enrichment tests.

## Methods

### RNA-seq data analysis

We analyzed 35 publicly available human or mouse RNA-seq datasets from GEO [30] (S1 Table). We sought datasets that were (1) published in recent years (mostly in 2017–18), (2) contained 2–4 replicate samples of each biological condition, (3) probed treatments with well-documented biological responses (e.g., TNFα) to ease functional interpretation and recognition of true calls by GSEA, and (4) collectively covered diverse biological processes. We downloaded either raw count data files when provided by GEO or, otherwise, raw sequence fastq files (from SRA DB). In the latter, reads were aligned to the reference genome (hg19 for Hs and mm10 for Mm) using TopHat2 [31], and gene count data were generated using FeatureCounts [32]. We calculated cpm levels, and in each dataset analysis, we included only the expressed genes (defined as those whose expression was at least 1.0 cpm in all replicate samples of at least one of the biological condition probed in the dataset). Following this filtering step, gene counts were normalized using six different normalization methods: RPKM [7], RPKM followed by quantile normalization [25], TMM [8], RLE [9], RLE followed by RPKM, and UQ normalization followed by RPKM [11], all implemented in edgeR [33]. cqn and EDASeq (both available as Bioconductor packages) were applied to expression count data. Gene-expression FC was either calculated by dividing normalized expression levels (after adding 1.0 to both numerator and denominator and averaging over replicate samples in the treatment versus control comparisons) or estimated by edgeR regression model fit. Gene annotations were downloaded from GENCODE (v25 for Hs and vM10 for Mm) [34]. For genes with multiple transcripts, we took the length of the principal transcript (as defined by GENCODE's annotation of principal and alternative splice isoforms [APPRIS] annotations [35]) or the length of the longest transcript if principal transcript is not defined for the gene. All statistical analyses were performed in R. Statistical significance of Spearman's correlation was calculated using the cor.test function.

Our R script and raw counts data for the RNA-seq datasets analyzed in this study are provided at https://github.com/ElkonLab/RNA-seq_length_bias.

The GSEAs that we carried out in this study used the pre-ranked GSEA method (GseaPreranked function; gsea v2.2.2). We ran CAMERA, implemented in the limma package (v3.38.3) [36], using either inter.gene.cor = NA to get gene-correlation estimates for each gene set or inter.gene.cor = 0 to run the tests without accounting for intergene correlations.

## Supporting information

S1 Fig Recurrent coupling between differential gene expression and gene length in RNA-seq data. Relationship between gene-expression FC and length in 35 publicly available RNA-seq datasets. For each dataset, normalized expression levels were averaged over replicate samples, and FC was calculated as (log2) ratio of these means (the number of replicates per condition in each dataset is given in S1 Table). Results shown here are based on RPKM normalization. Very similar results were obtained using five alternative normalization methods (S1 Table). Data underlying the results presented in this figure are provided in https://github.com/ElkonLab/RNA-seq_length_bias/. FC, fold change; RNA-seq, RNA sequencing; RPKM, reads per kilobase of transcript length per million reads.
(PDF)

S2 Fig Sample-specific technical length effect in RNA-seq experiments couples gene length and differential expression. Relationship between gene length and expression FC in comparisons between individual replicate samples from 35 publicly available RNA-seq datasets. For each dataset, the replicates pair that showed the strongest length–FC coupling is shown. Results shown here are based on RPKM normalization. Very similar results were obtained using five alternative normalization methods (S2 Table). Data underlying the results presented in this figure are provided in https://github.com/ElkonLab/RNA-seq_length_bias/. FC, fold change; RNA-seq, RNA sequencing; RPKM, reads per kilobase of transcript length per million reads.
(PDF)

S3 Fig Sample-specific length bias leads to false positive results by GSEA that are canceled by cqn. (A-N) All items in this figure show results for comparisons between replicate samples of the same biological condition. (The biological condition and the GEO dataset are indicated in the title of each item. Dataset numbers refer to S1 Table, and GEO IDs of the replicate samples are given in S2 Table.) Each item shows a certain gene set that is detected as enriched by GSEA when applied to the original dataset (left) and shows the removal of the FC–length coupling by cqn (middle), similar to the presentation detailed in Fig 3. Importantly, in all cases, the false positive calls stemming from sample-specific length biases were canceled by cqn (right). Data underlying the results presented in this figure are provided in https://github.com/ElkonLab/RNA-seq_length_bias/. cqn, conditional quantile normalization; FC, fold change; GEO, Gene Expression Omnibus; GSEA, gene-set enrichment analysis.
(PDF)

S4 Fig Sample-specific length bias is not corrected by a sample specific GC-content covariate. (A) (Top left) Sample-specific length bias in the TNFa (GSE64233) dataset. This figure is the same as Fig 1A, showing the comparison between treated and control samples. FC is strongly linked to gene length. (Bottom left) This dataset shows only very minimal sample specific GC bias, indicating that sample-specific length bias is not a mere reflection of GC effects. (Top right) Running cqn with only GC content as sample-specific covariate did not remove the length effect in this dataset. (Bottom right) Adding the sample-specific length covariate to the cqn run completely corrects the length bias. (B) Analysis of the same dataset using EDASeq. (Top) EDASeq run with FQ between-samples normalization and no within-samples normalization shows the absence of GC-content effect (left) and strong length bias (right) in this dataset. (Middle) EDASeq run with within-samples normalization adjusting for GC content followed by FQ between-samples normalization does not remove the length bias. (Bottom) EDASeq run with within-samples normalization adjusting for gene length followed by FQ between-samples normalization effectively corrects the length bias. See S4 Table for results on the 35 RNA-seq datasets. Data underlying the results presented in this figure are provided in https://github.com/ElkonLab/RNA-seq_length_bias/. cqn, conditional quantile normalization; FC, fold change; FQ, full quantile; RNA-seq, RNA sequencing; TNFa, tumor necrosis alpha.
(PDF)

S5 Fig Sample-specific length bias correction by cqn reduces GSEA false positive calls without compromising detection of true ones. (A-G). Each item shows GSEA results on the original dataset (left) and after the application of cqn (right). The enrichment of the upper gene set is biologically genuine, whereas the enrichment of the bottom gene set is an artifact caused by sample-specific length bias and is canceled by cqn. Data underlying the results presented in this figure are provided in https://github.com/ElkonLab/RNA-seq_length_bias/. cqn, conditional quantile normalization; GSEA, gene-set enrichment analysis.
(PDF)

S1 Table Analysis of gene length–FC relationship between treatment and control samples in 35 RNA-seq datasets. FC, fold change; RNA-seq, RNA sequencing.
(XLSX)

S2 Table Analysis of gene length–FC relationship between replicate samples in 35 RNA-seq datasets. S2A Table contains results for the replicate pairs showing the strongest length effect in each dataset, and S2B Table shows the results for all pairwise comparisons. FC, fold change; RNA-seq, RNA sequencing.
(XLSX)

S3 Table Analysis of GC bias in the 35 RNA-seq datasets. RNA-seq, RNA sequencing.
(XLSX)

S4 Table Sample-specific length bias correction by cqn and EDASeq. cqn, conditional normalized quantile. (XLSX)

S1 Data TNFα dataset (GSE64233)—raw counts and normalized data. TNFα, tumor necrosis factor alpha.
(XLSX)

S2 Data TNFα dataset—cqn-normalized data and FC estimates. FC, fold change; TNFα, tumor necrosis factor alpha.
(XLSX)

S3 Data EMT dataset (GSE114572)—cqn-normalized data and FC estimates. cqn, conditional normalized quantile; EMT, epithelial–mesenchymal transition; FC, fold change.
(XLSX)

## Abbreviations

CAMERA: Correlation Adjusted MEan RAnk gene-set test
cqn: conditional quantile normalization
DEG: differentially expressed gene
ECM: extracellular matrix
EMT: epithelial–mesenchymal transition
FC: fold change
FDR: false discovery rate
GSEA: gene-set enrichment analysis
lowess: locally weighted scatterplot smoothing
NES: normalized enrichment score
RLE: relative log expression
RNA: seq, RNA sequencing
RPKM: reads per kilobase of transcript length per million reads
TMM: Trimmed Mean of M values
TNF: tumor necrosis factor
UQ: upper quartile
