# Supplementary material for: Recurrent functional misinterpretation of RNA-seq data caused by sample-specific gene length bias
Source: PLoS Biol. 2019 Nov 12;17(11):e3000481. doi: 10.1371/journal.pbio.3000481 (PMC6850523; doi:10.1371/journal.pbio.3000481)
Supplement: S1 Fig — Relationship between gene-expression FC and length in 35 publicly available RNA-seq datasets. For each dataset, normalized expression levels were averaged over replicate samples, and FC was calculated as (log2) ratio of these means (the number of replicates per condition in each dataset is given in S1 Table). Results shown here are based on RPKM normalization. Very similar results were obtained using five alternative normalization methods (S1 Table). Data underlying the results presented in this figure are provided in https://github.com/ElkonLab/RNA-seq_length_bias/. FC, fold change; RNA-seq, RNA sequencing; RPKM, reads per kilobase of transcript length per million reads. (PDF) [file pbio.3000481.s001.pdf]

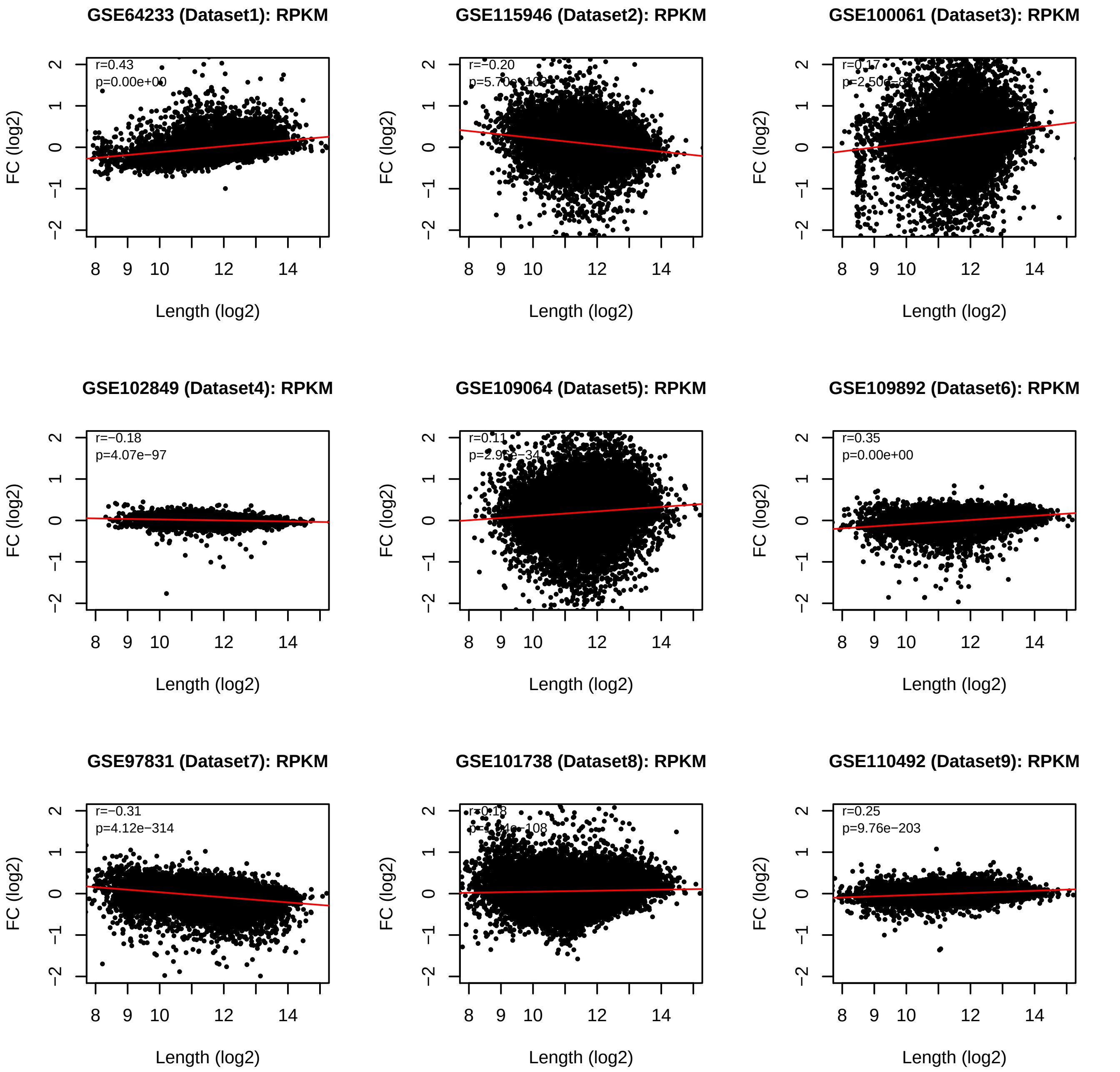

GSE64233 (Dataset1): RPKM
r=0.43
p=0.00e+00
GSE115946 (Dataset2): RPKM
r=−0.20
GSE100061 (Dataset3): RPKM
r=0.17
GSE102849 (Dataset4): RPKM
r=−0.18
p=4.07e−97
GSE109064 (Dataset5): RPKM
r=0.11
GSE109892 (Dataset6): RPKM
r=0.35
p=0.00e+00
GSE97831 (Dataset7): RPKM
r=−0.31
p=4.12e−314
GSE101738 (Dataset8): RPKM
r=0.18
GSE110492 (Dataset9): RPKM
r=0.25
p=9.76e−203
FC (log2)
Length (log2)

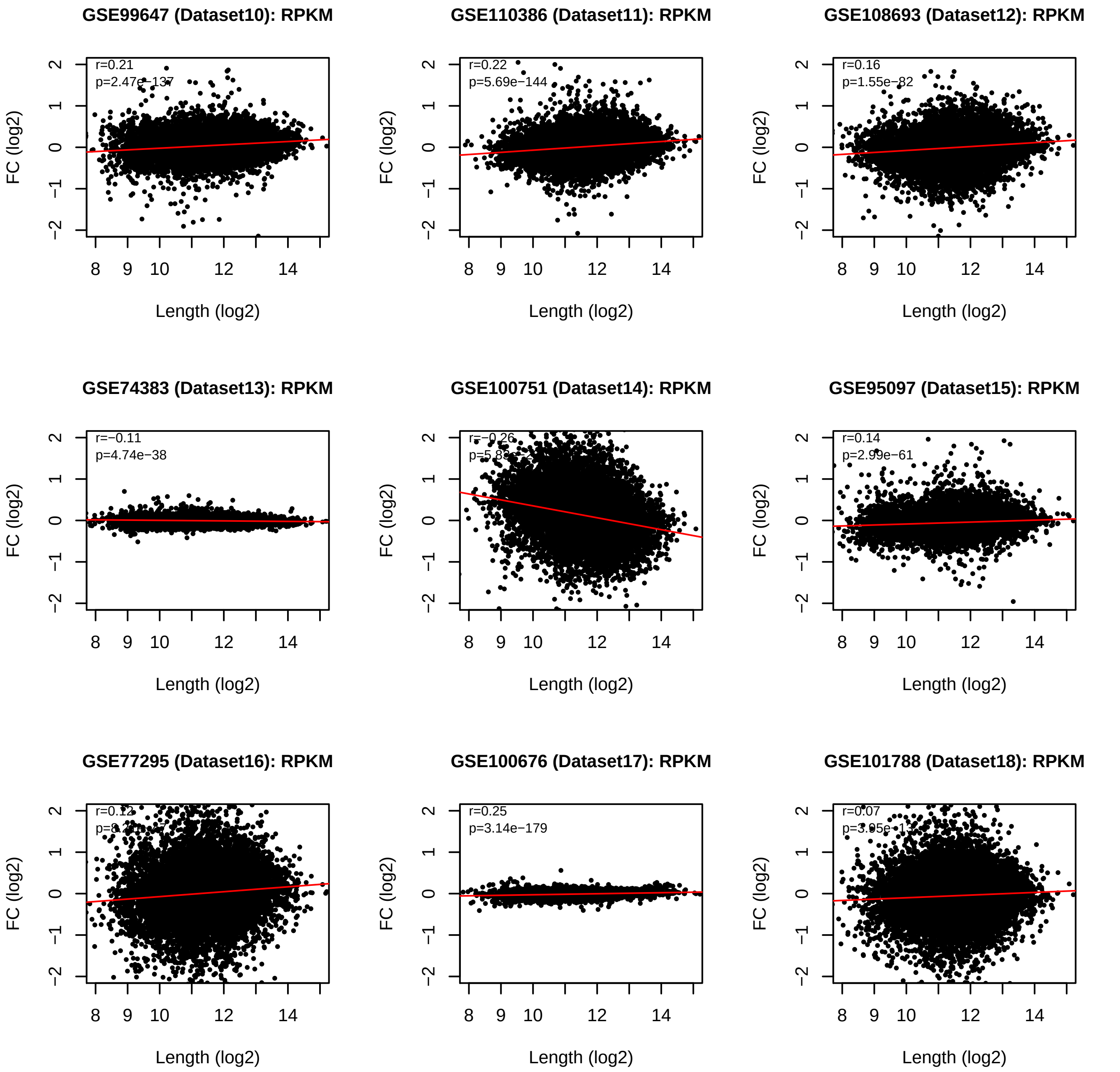
GSE99647 (Dataset10): RPKM
r=0.21
p=2.47e−137
GSE110386 (Dataset11): RPKM
r=0.22
p=5.69e−144
GSE108693 (Dataset12): RPKM
r=0.16
p=1.55e−82
GSE74383 (Dataset13): RPKM
r=−0.11
p=4.74e−38
GSE100751 (Dataset14): RPKM
r=−0.26
GSE95097 (Dataset15): RPKM
r=0.14
p=2.99e−61
GSE77295 (Dataset16): RPKM
GSE100676 (Dataset17): RPKM
r=0.25
p=3.14e−179
GSE101788 (Dataset18): RPKM
r=0.07
FC (log2)
Length (log2)

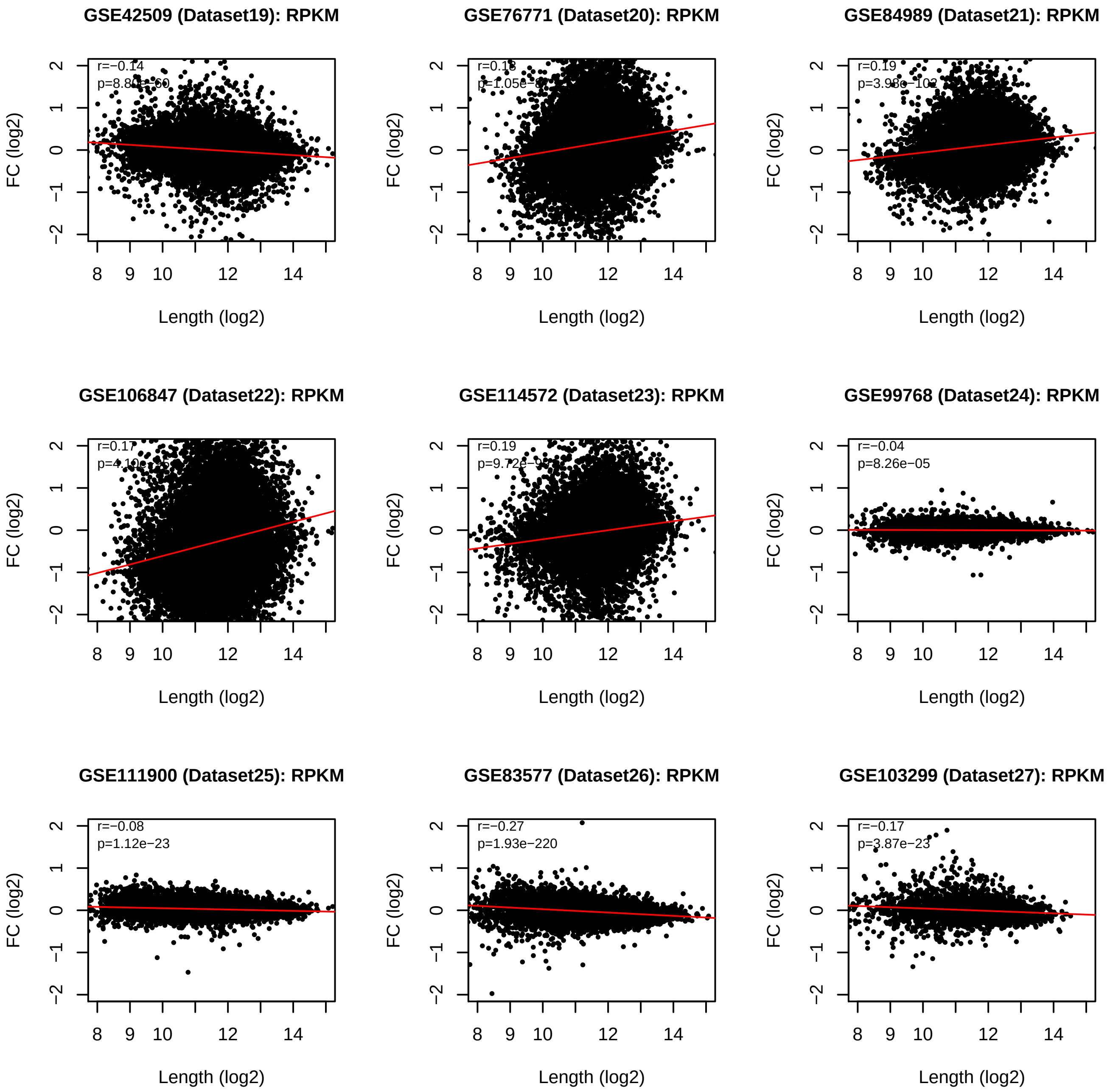

GSE42509 (Dataset19): RPKM
r=−0.14
GSE76771 (Dataset20): RPKM
GSE84989 (Dataset21): RPKM
r=0.19
GSE106847 (Dataset22): RPKM
GSE114572 (Dataset23): RPKM
r=0.19
GSE99768 (Dataset24): RPKM
r=−0.04
p=8.26e−05
GSE111900 (Dataset25): RPKM
r=−0.08
p=1.12e−23
GSE83577 (Dataset26): RPKM
r=−0.27
p=1.93e−220
GSE103299 (Dataset27): RPKM
r=−0.17
FC (log2)
Length (log2)

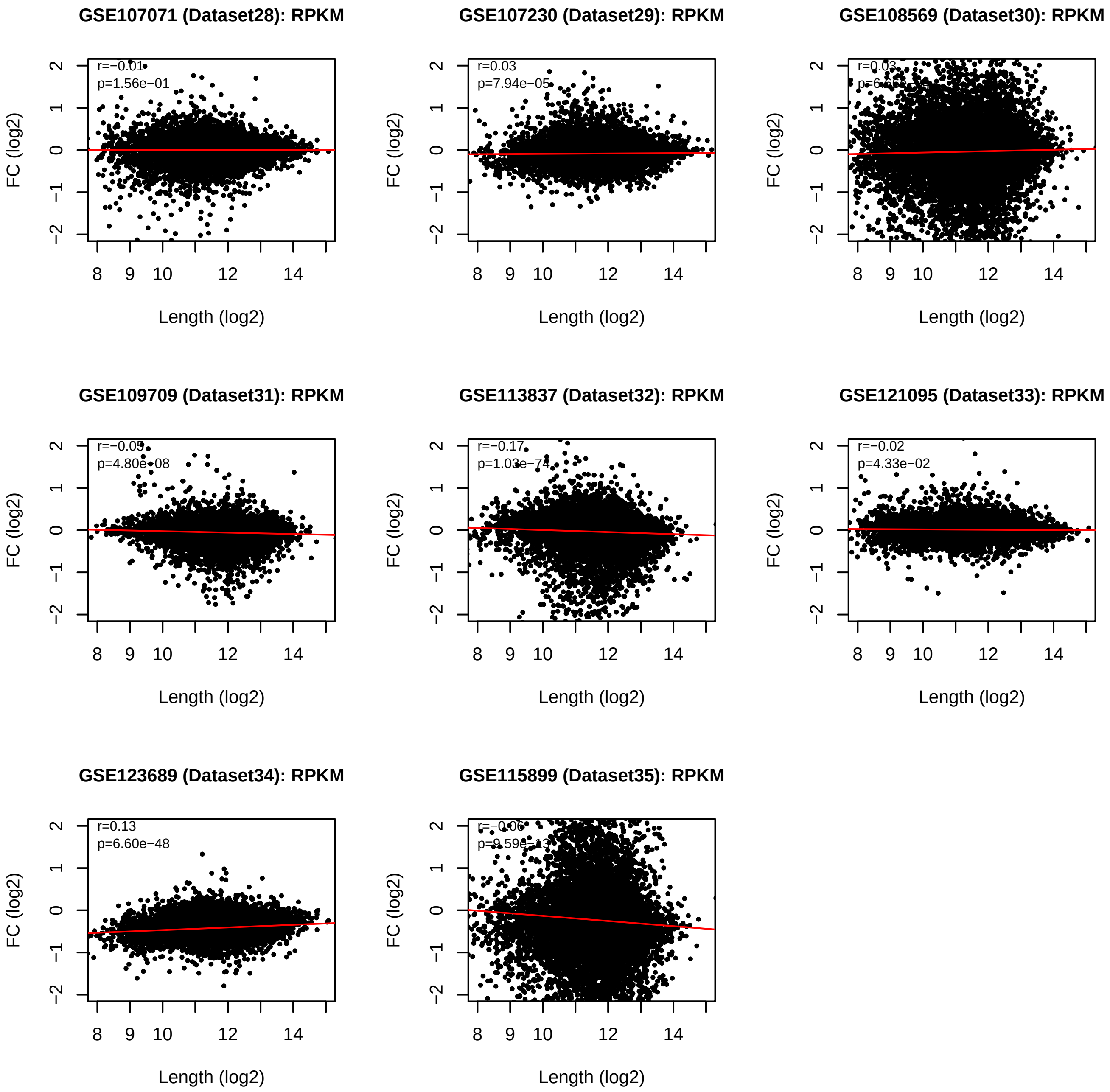
GSE107071 (Dataset28): RPKM
r=−0.01
p=1.56e−01
GSE107230 (Dataset29): RPKM
r=0.03
p=7.94e−05
GSE108569 (Dataset30): RPKM
r=0.03
GSE109709 (Dataset31): RPKM
r=−0.05
p=4.80e−08
GSE113837 (Dataset32): RPKM
r=−0.17
p=1.03e−74
GSE121095 (Dataset33): RPKM
r=−0.02
p=4.33e−02
GSE123689 (Dataset34): RPKM
r=0.13
p=6.60e−48
GSE115899 (Dataset35): RPKM
r=−0.06
FC (log2)
Length (log2)
2
1
0
−1
−2
8
9
10
12
14
