# Supplementary material for: Recurrent functional misinterpretation of RNA-seq data caused by sample-specific gene length bias
Source: PLoS Biol. 2019 Nov 12;17(11):e3000481. doi: 10.1371/journal.pbio.3000481 (PMC6850523; doi:10.1371/journal.pbio.3000481)
Supplement: S2 Fig — Relationship between gene length and expression FC in comparisons between individual replicate samples from 35 publicly available RNA-seq datasets. For each dataset, the replicates pair that showed the strongest length–FC coupling is shown. Results shown here are based on RPKM normalization. Very similar results were obtained using five alternative normalization methods (S2 Table). Data underlying the results presented in this figure are provided in https://github.com/ElkonLab/RNA-seq_length_bias/. FC, fold change; RNA-seq, RNA sequencing; RPKM, reads per kilobase of transcript length per million reads. (PDF) [file pbio.3000481.s002.pdf]

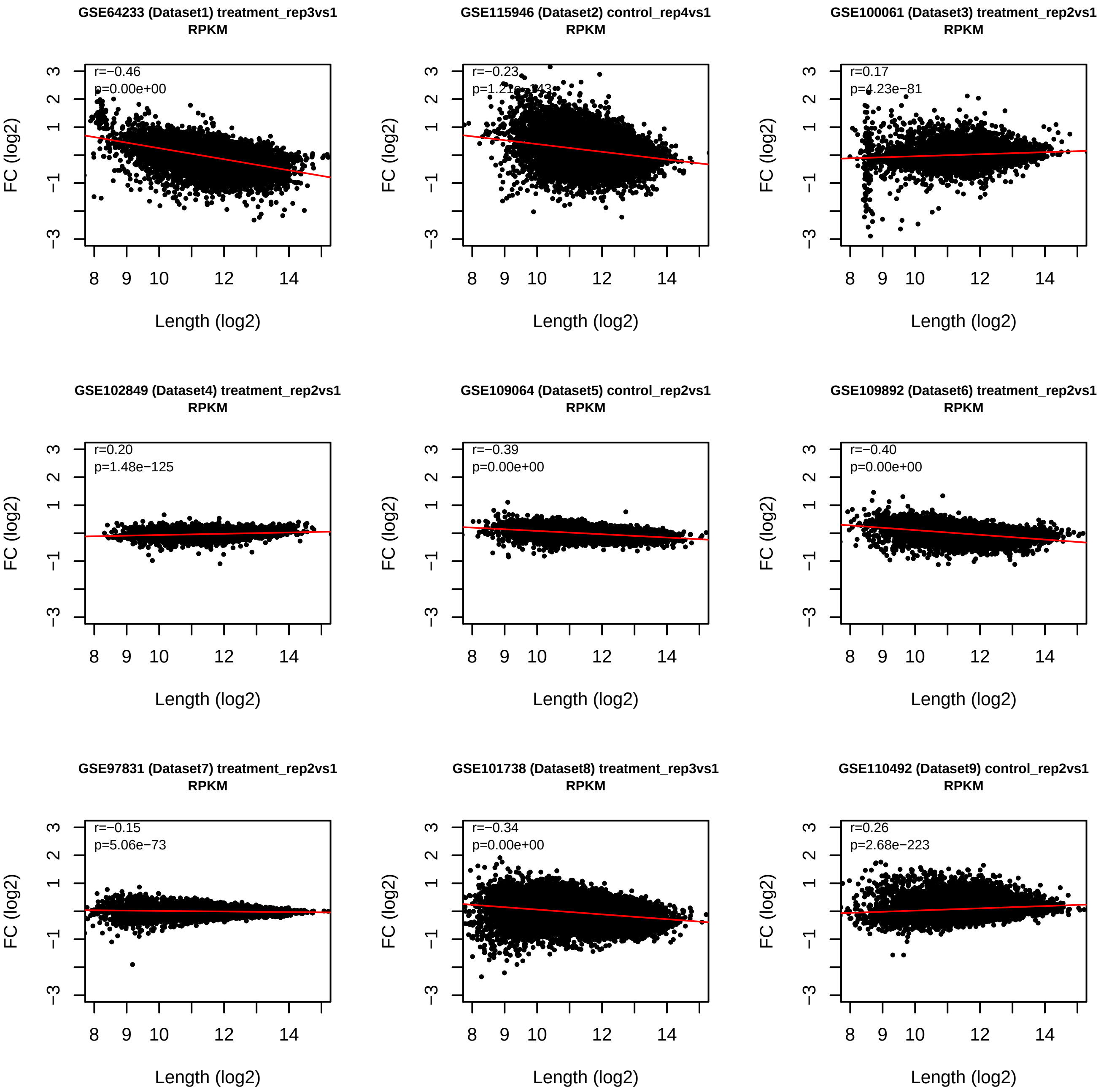
GSE64233 (Dataset1) treatment_rep3vs1
RPKM
r=−0.46
p=0.00e+00
GSE115946 (Dataset2) control_rep4vs1
RPKM
r=−0.23
p=1.21e−143
GSE100061 (Dataset3) treatment_rep2vs1
RPKM
r=0.17
p=4.23e−81
GSE102849 (Dataset4) treatment_rep2vs1
RPKM
r=0.20
p=1.48e−125
GSE109064 (Dataset5) control_rep2vs1
RPKM
r=−0.39
p=0.00e+00
GSE109892 (Dataset6) treatment_rep2vs1
RPKM
r=−0.40
p=0.00e+00
GSE97831 (Dataset7) treatment_rep2vs1
RPKM
r=−0.15
p=5.06e−73
GSE101738 (Dataset8) treatment_rep3vs1
RPKM
r=−0.34
p=0.00e+00
GSE110492 (Dataset9) control_rep2vs1
RPKM
r=0.26
p=2.68e−223
FC (log2)
Length (log2)

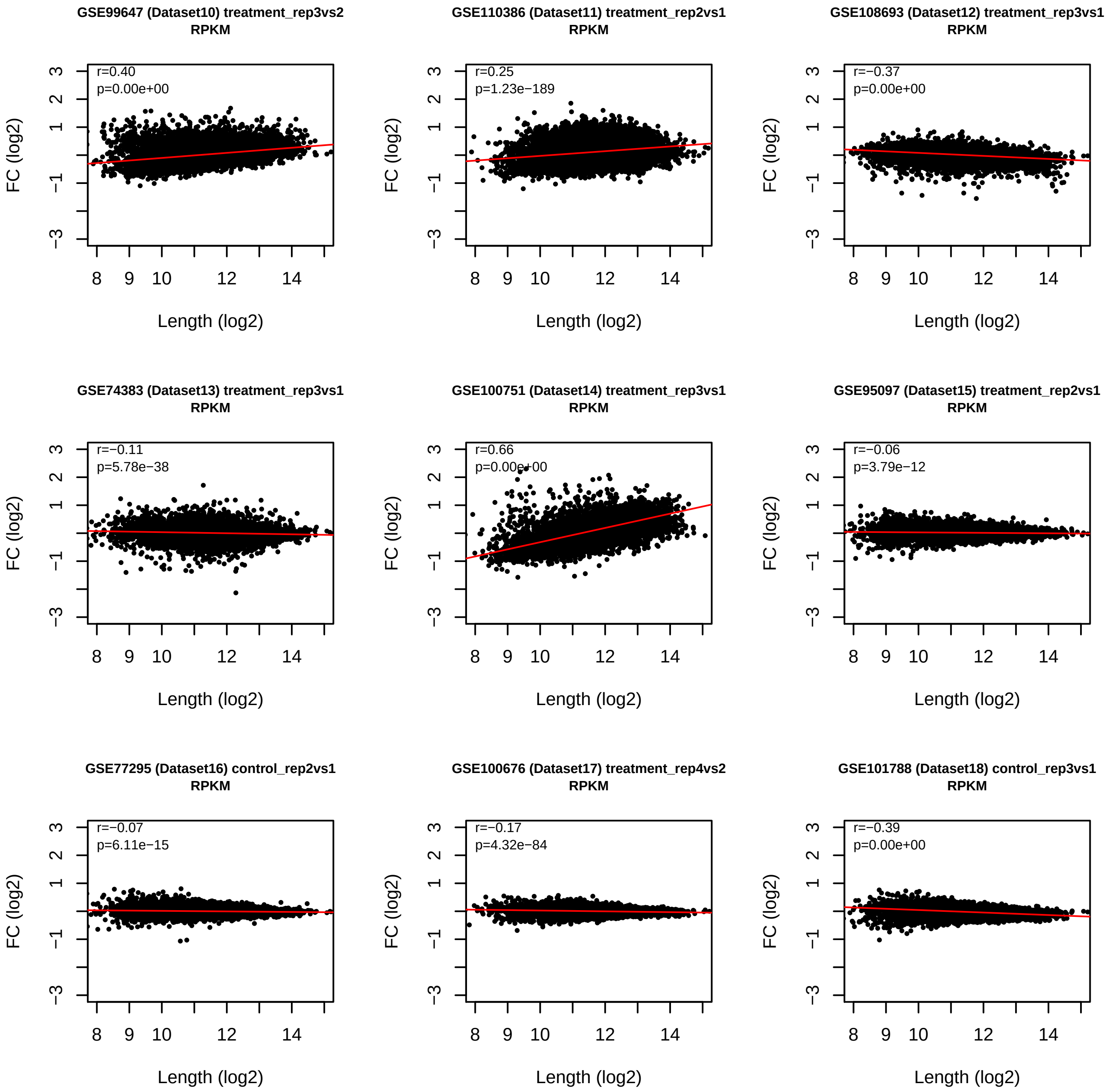
GSE99647 (Dataset10) treatment_rep3vs2
RPKM
r=0.40
p=0.00e+00
GSE110386 (Dataset11) treatment_rep2vs1
RPKM
r=0.25
p=1.23e−189
GSE108693 (Dataset12) treatment_rep3vs1
RPKM
r=−0.37
p=0.00e+00
GSE74383 (Dataset13) treatment_rep3vs1
RPKM
r=−0.11
p=5.78e−38
GSE100751 (Dataset14) treatment_rep3vs1
RPKM
r=0.66
p=0.00e+00
GSE95097 (Dataset15) treatment_rep2vs1
RPKM
r=−0.06
p=3.79e−12
GSE77295 (Dataset16) control_rep2vs1
RPKM
r=−0.07
p=6.11e−15
GSE100676 (Dataset17) treatment_rep4vs2
RPKM
r=−0.17
p=4.32e−84
GSE101788 (Dataset18) control_rep3vs1
RPKM
r=−0.39
p=0.00e+00
FC (log2)
Length (log2)
3
2
1
−1
−3
8
9
10
12
14

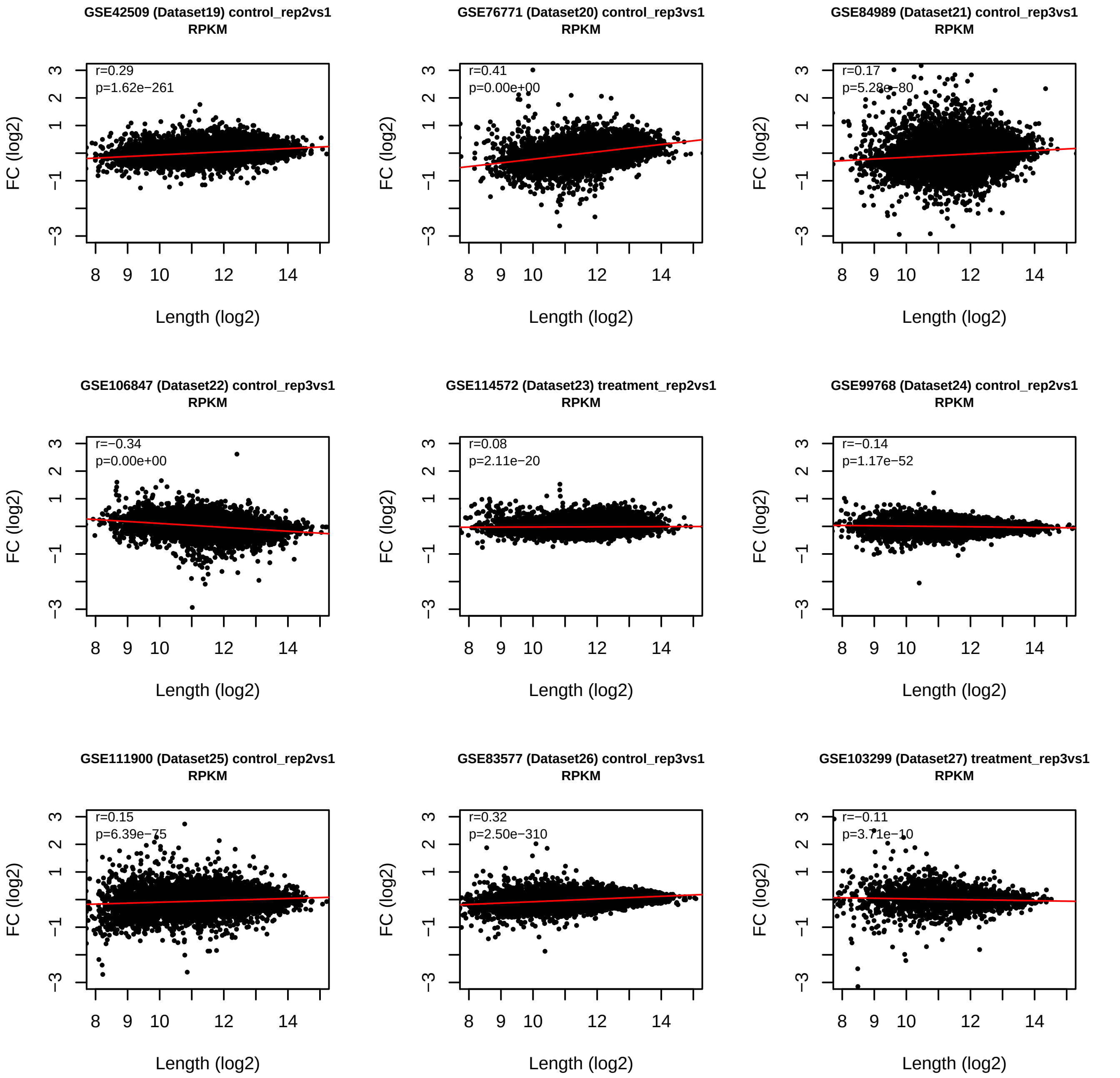
GSE42509 (Dataset19) control_rep2vs1
RPKM
r=0.29
p=1.62e−261
GSE76771 (Dataset20) control_rep3vs1
RPKM
r=0.41
p=0.00e+00
GSE84989 (Dataset21) control_rep3vs1
RPKM
r=0.17
p=5.28e−80
GSE106847 (Dataset22) control_rep3vs1
RPKM
r=−0.34
p=0.00e+00
GSE114572 (Dataset23) treatment_rep2vs1
RPKM
r=0.08
p=2.11e−20
GSE99768 (Dataset24) control_rep2vs1
RPKM
r=−0.14
p=1.17e−52
GSE111900 (Dataset25) control_rep2vs1
RPKM
r=0.15
p=6.39e−75
GSE83577 (Dataset26) control_rep3vs1
RPKM
r=0.32
p=2.50e−310
GSE103299 (Dataset27) treatment_rep3vs1
RPKM
r=−0.11
p=3.71e−10
FC (log2)
Length (log2)

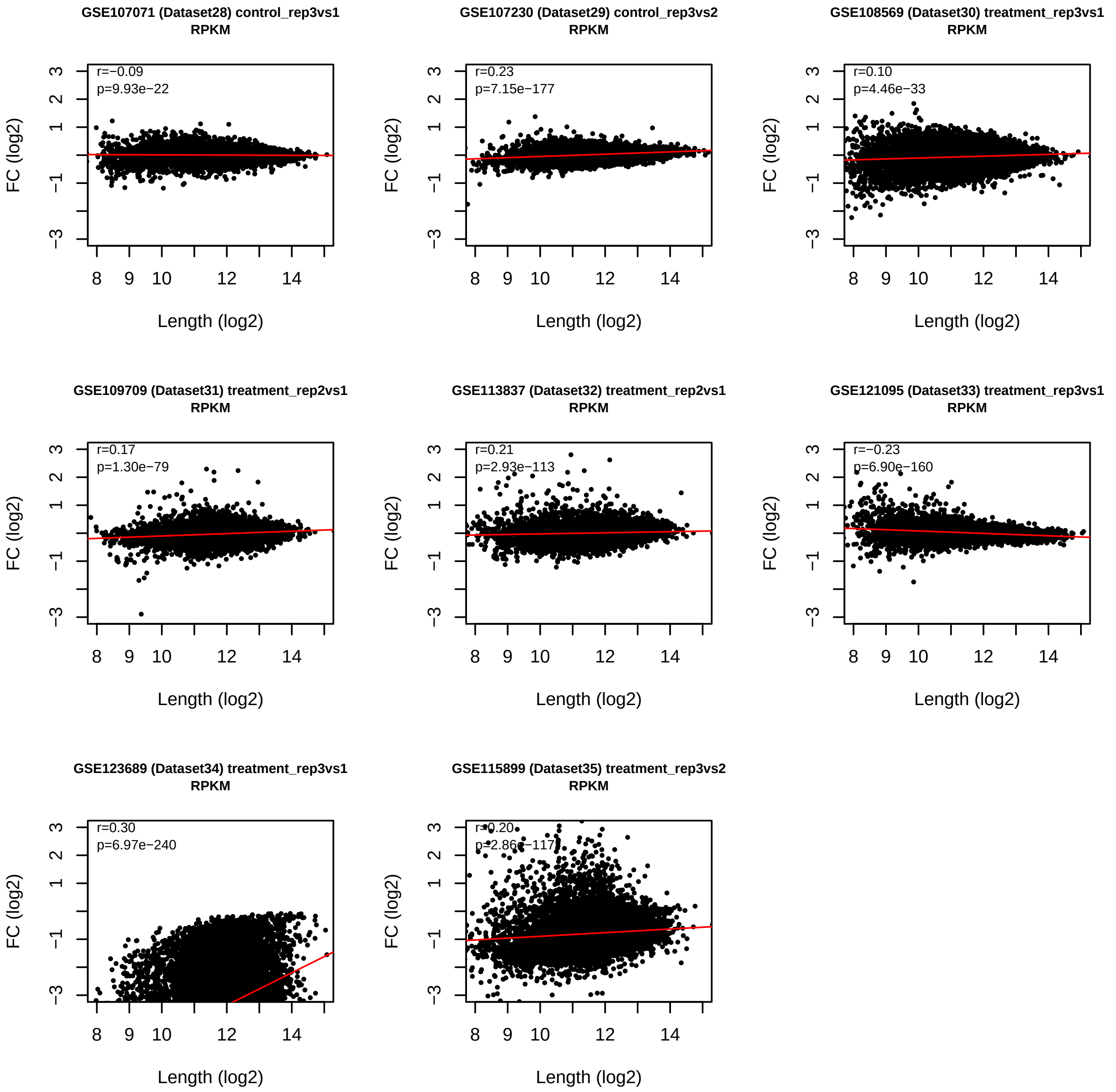
GSE107071 (Dataset28) control_rep3vs1
RPKM
r=−0.09
p=9.93e−22
GSE107230 (Dataset29) control_rep3vs2
RPKM
r=0.23
p=7.15e−177
GSE108569 (Dataset30) treatment_rep3vs1
RPKM
r=0.10
p=4.46e−33
GSE109709 (Dataset31) treatment_rep2vs1
RPKM
r=0.17
p=1.30e−79
GSE113837 (Dataset32) treatment_rep2vs1
RPKM
r=0.21
p=2.93e−113
GSE121095 (Dataset33) treatment_rep3vs1
RPKM
r=−0.23
p=6.90e−160
GSE123689 (Dataset34) treatment_rep3vs1
RPKM
r=0.30
p=6.97e−240
GSE115899 (Dataset35) treatment_rep3vs2
RPKM
r=0.20
p=2.86e−117
FC (log2)
Length (log2)
