# Supplementary material for: Recurrent functional misinterpretation of RNA-seq data caused by sample-specific gene length bias
Source: PLoS Biol. 2019 Nov 12;17(11):e3000481. doi: 10.1371/journal.pbio.3000481 (PMC6850523; doi:10.1371/journal.pbio.3000481)
Supplement: S3 Fig — (A-N) All items in this figure show results for comparisons between replicate samples of the same biological condition. (The biological condition and the GEO dataset are indicated in the title of each item. Dataset numbers refer to S1 Table, and GEO IDs of the replicate samples are given in S2 Table.) Each item shows a certain gene set that is detected as enriched by GSEA when applied to the original dataset (left) and shows the removal of the FC–length coupling by cqn (middle), similar to the presentation detailed in Fig 3. Importantly, in all cases, the false positive calls stemming from sample-specific length biases were canceled by cqn (right). Data underlying the results presented in this figure are provided in https://github.com/ElkonLab/RNA-seq_length_bias/. cqn, conditional quantile normalization; FC, fold change; GEO, Gene Expression Omnibus; GSEA, gene-set enrichment analysis. (PDF) [file pbio.3000481.s003.pdf]

a Dataset #1: TNF (GSE64233): Replicate comparison

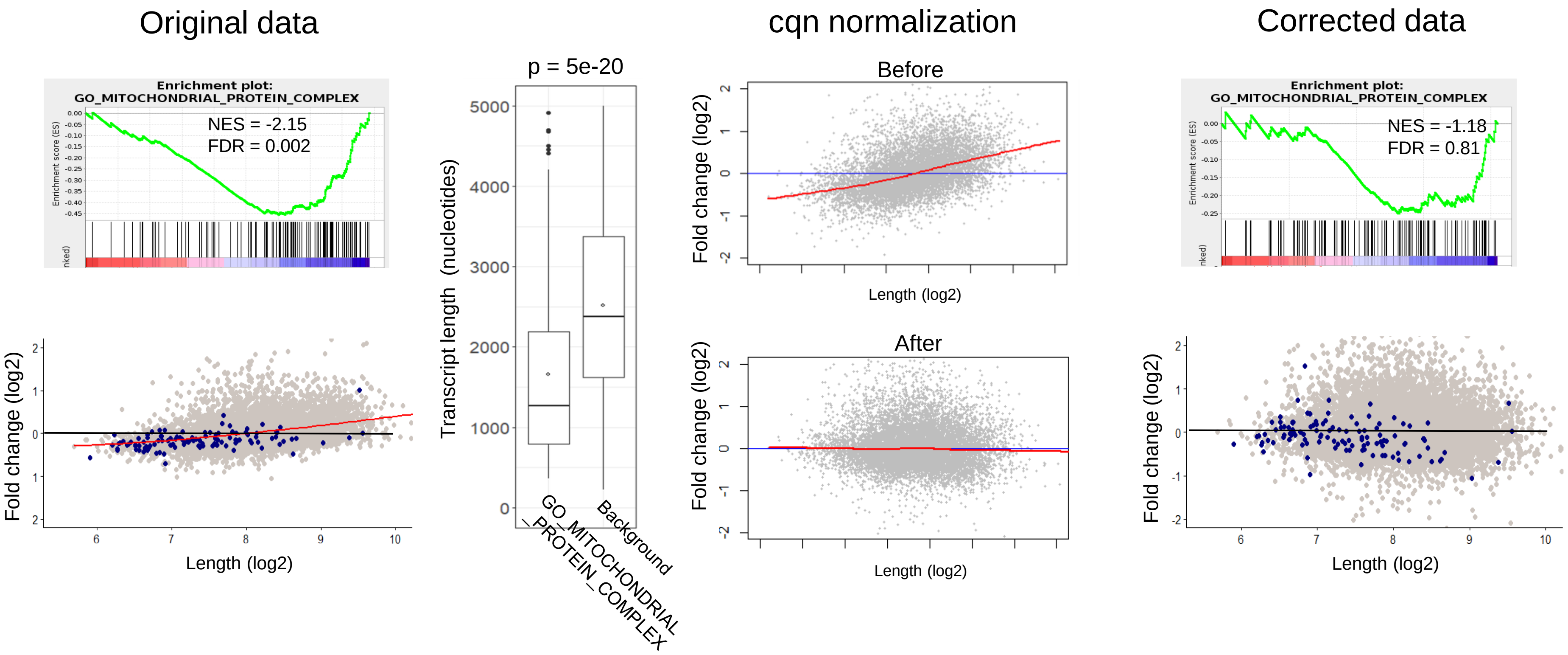

Supplementary Figure 3

b Dataset #1: TNF (GSE64233): Replicate comparison

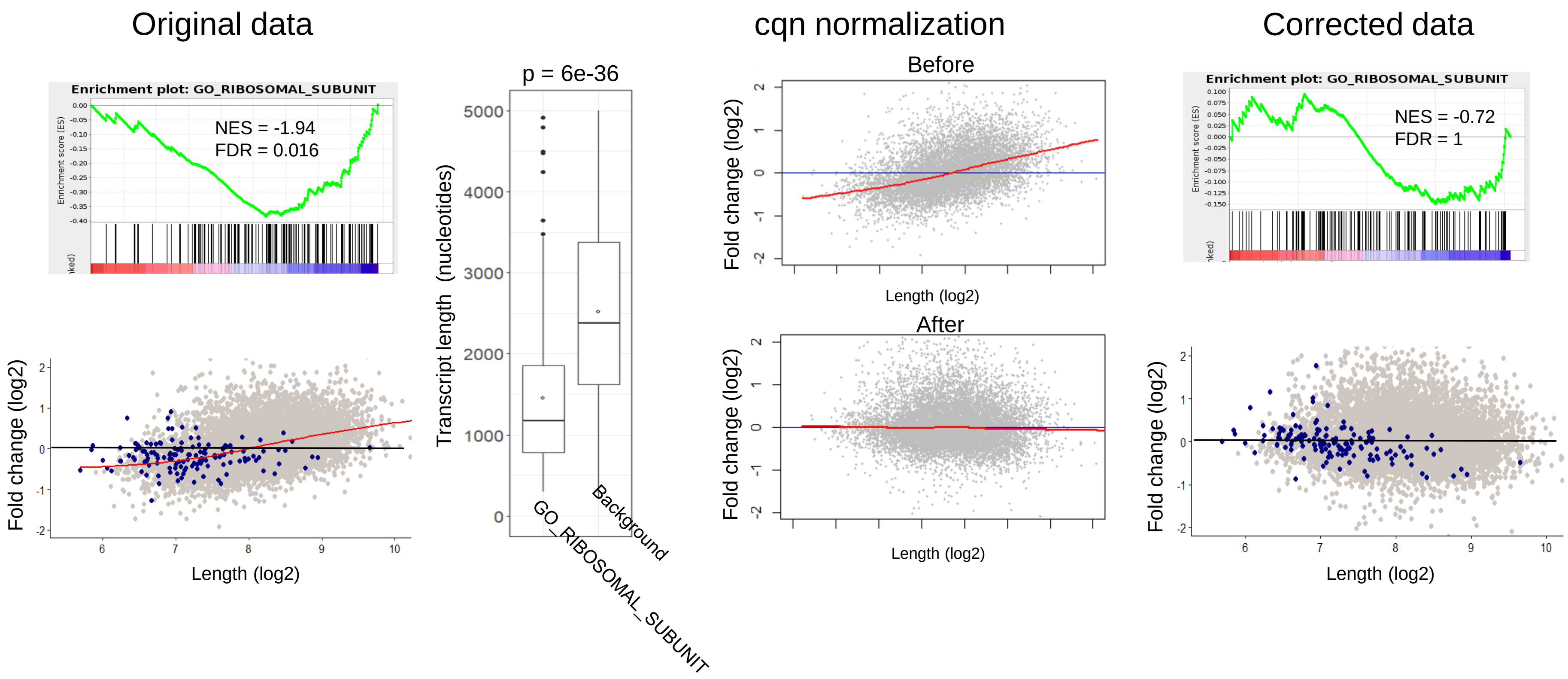

Supplementary Figure 3

C Dataset #1: TNF (GSE64233): Replicate comparison

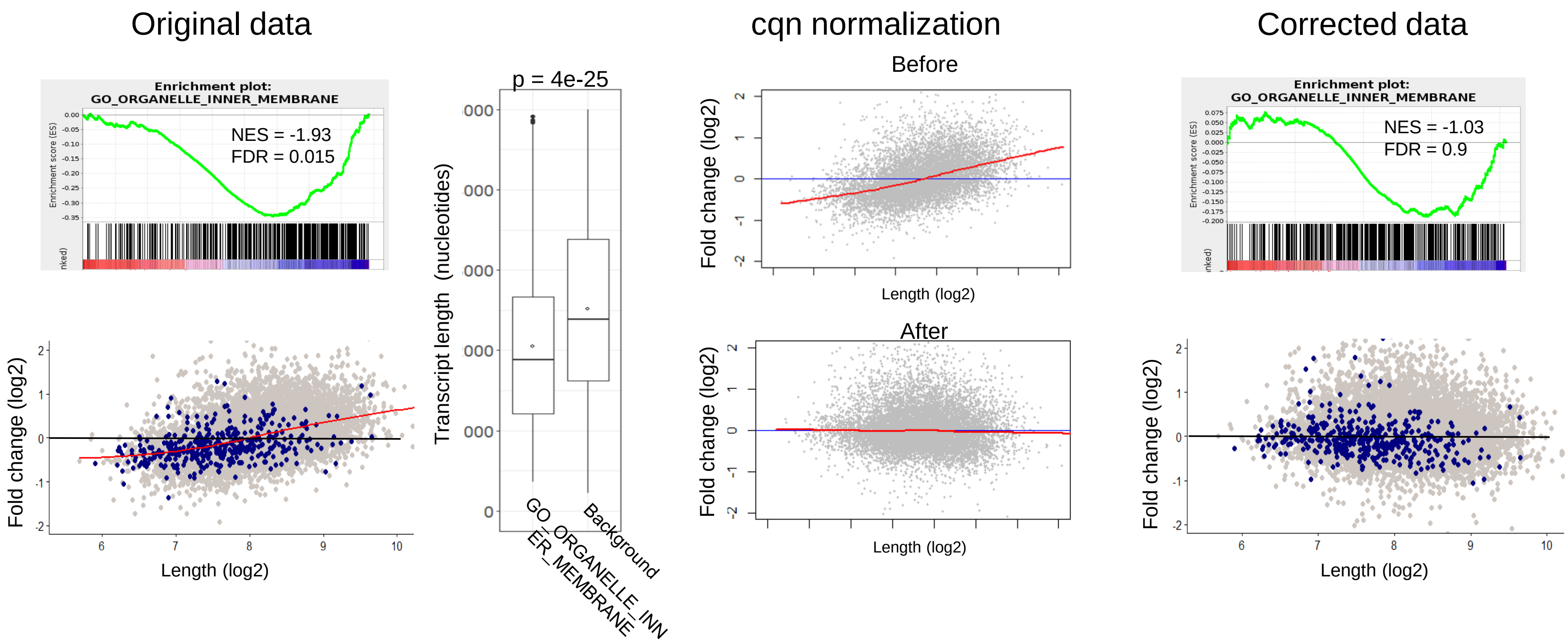

Supplementary Figure 3

d Dataset #10: shSRC-1 in BC cells (GSE99647): Replicate comparison

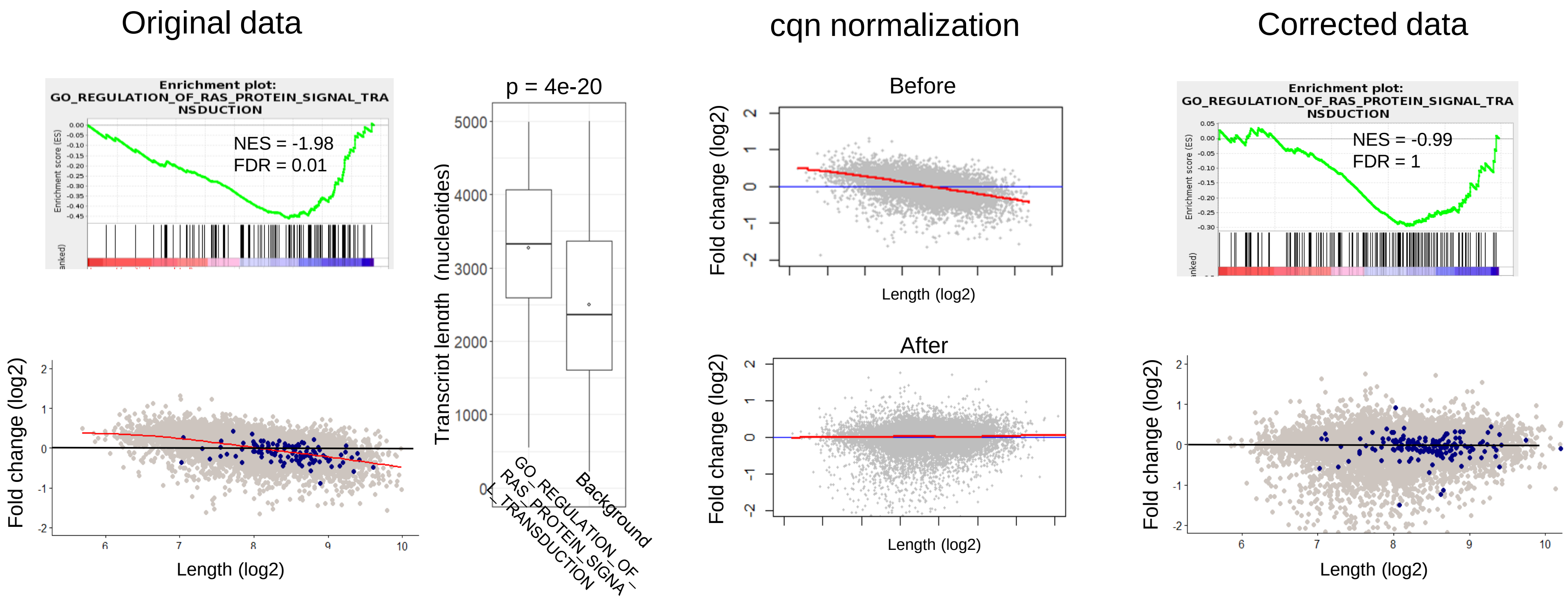

Supplementary Figure 3

e Dataset #10: shSRC-1 in BC cells (GSE99647): Replicate comparison

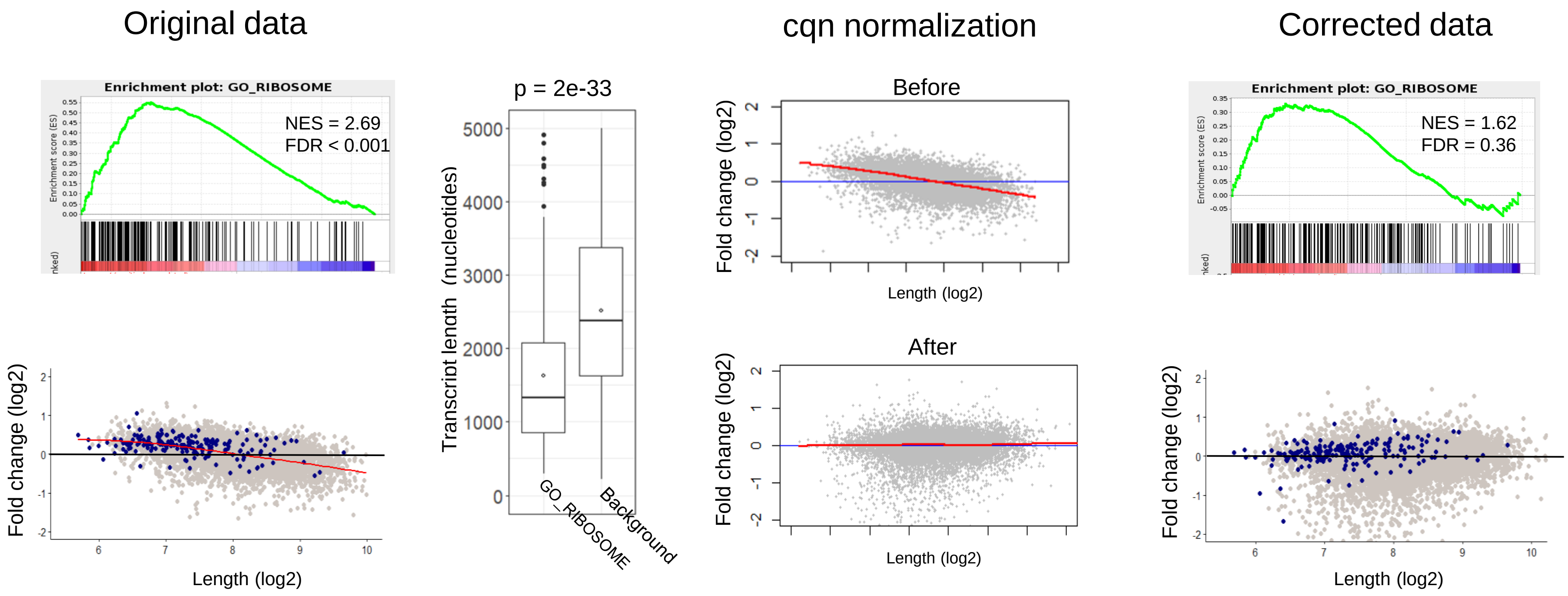

Supplementary Figure 3

f

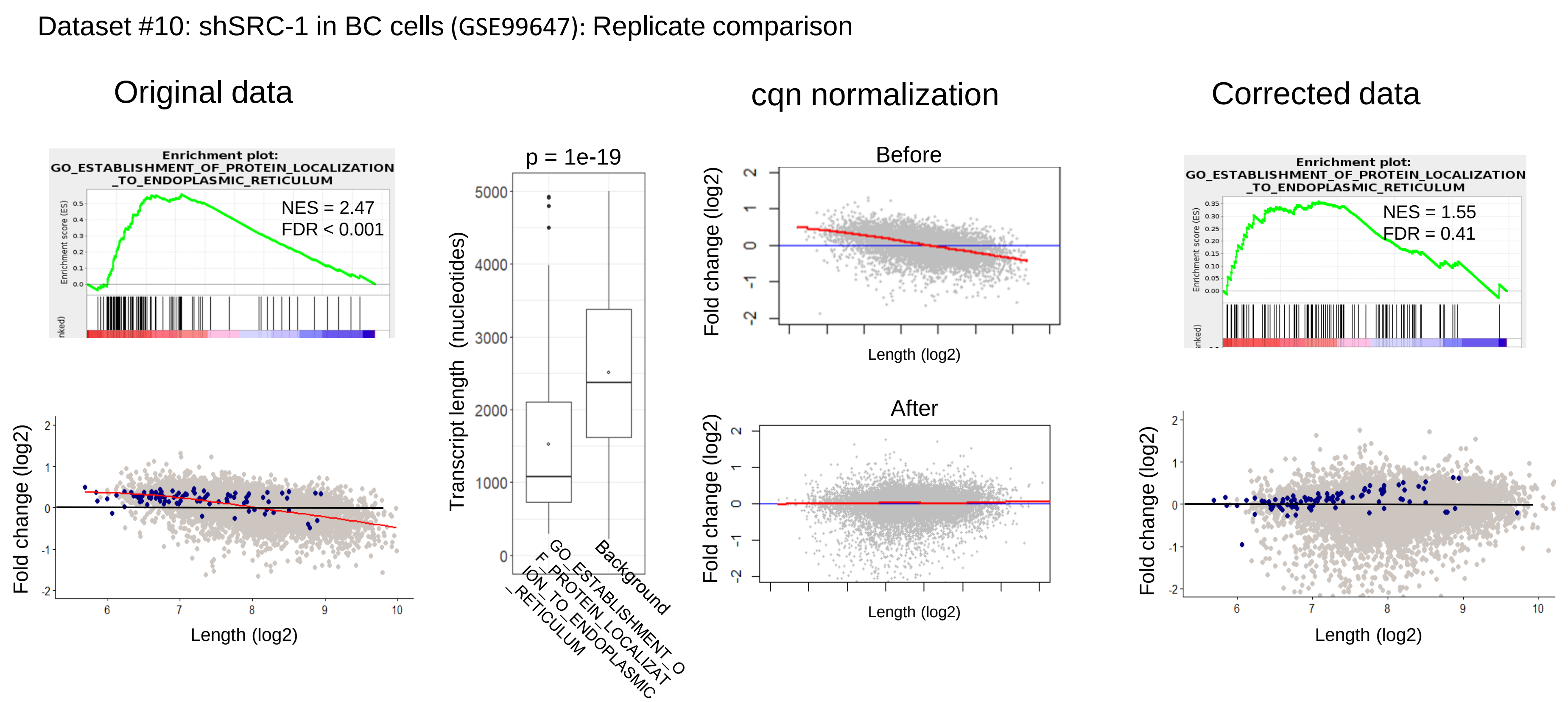

Supplementary Figure 3

g Dataset #10: shSRC-1 in BC cells (GSE99647): Replicate comparison

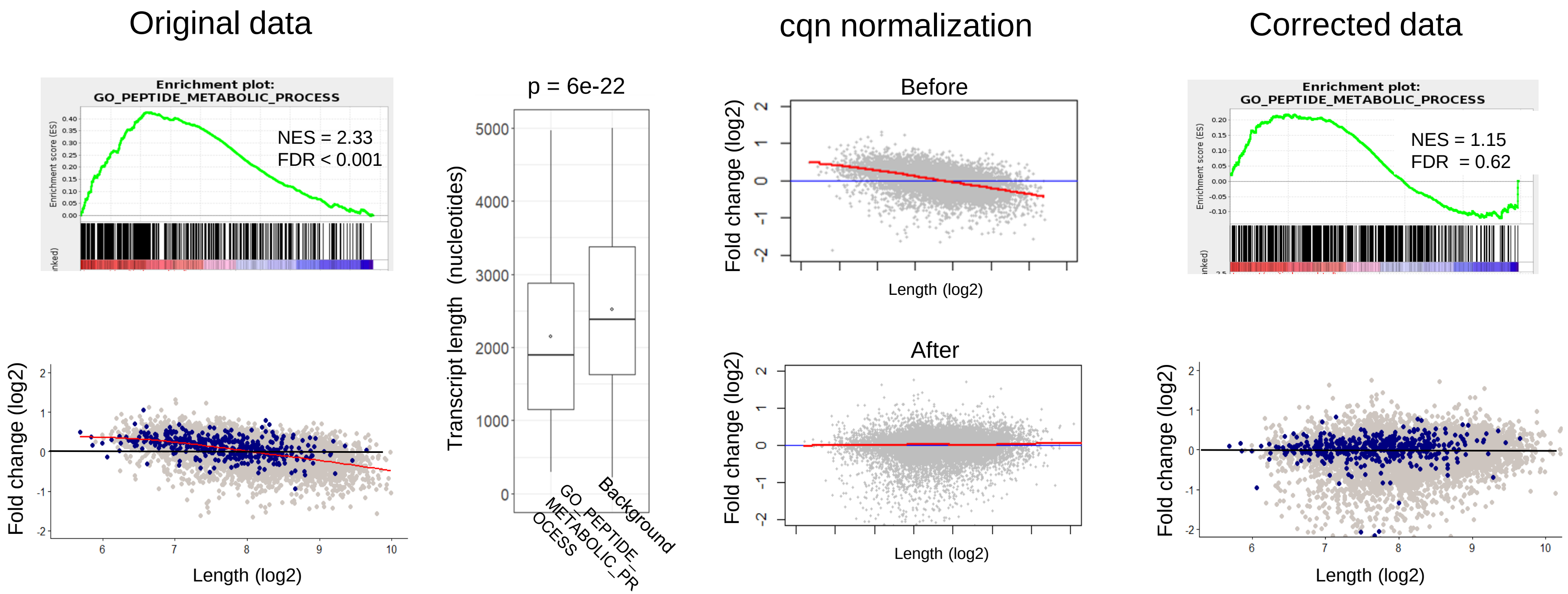

Supplementary Figure 3

h

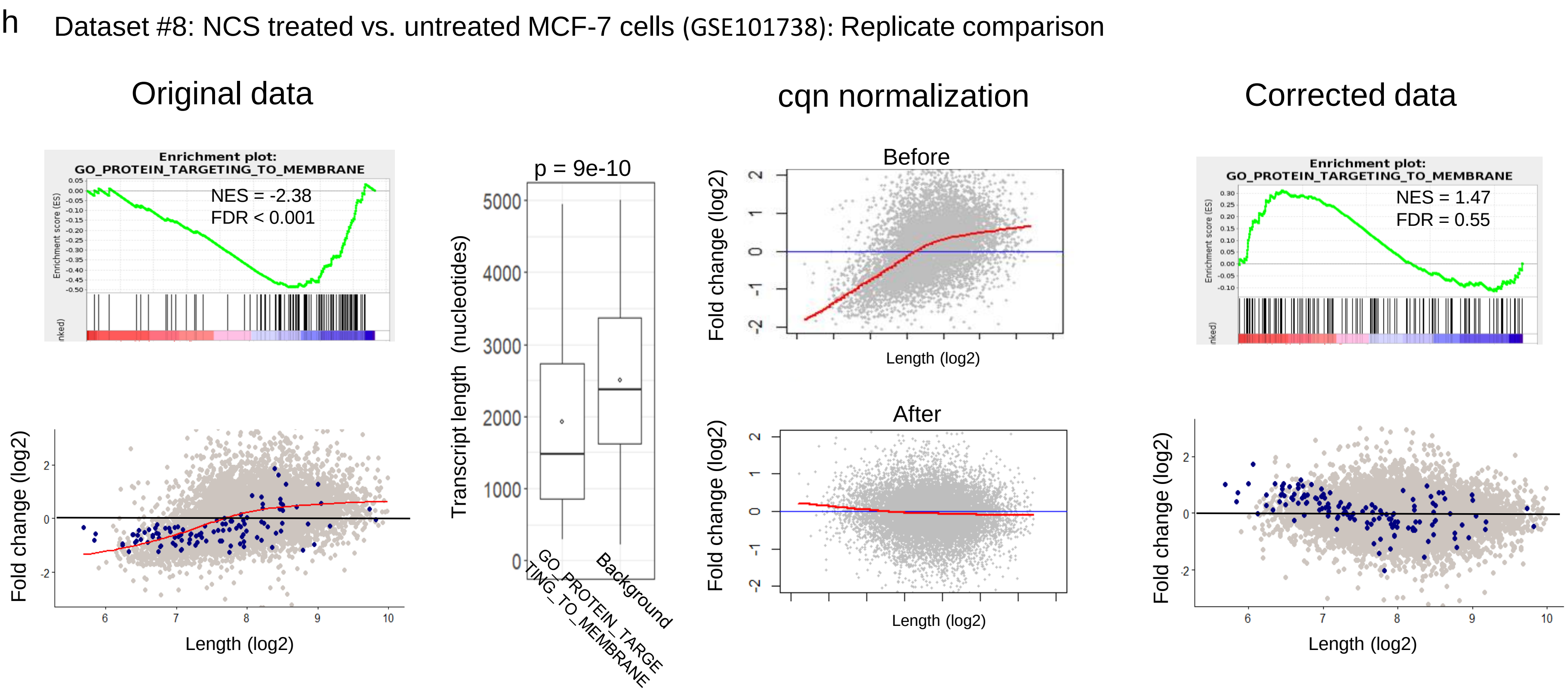

Supplementary Figure 3

i Dataset #8: NCS treated vs. untreated MCF-7 cells (GSE101738): Replicate comparison

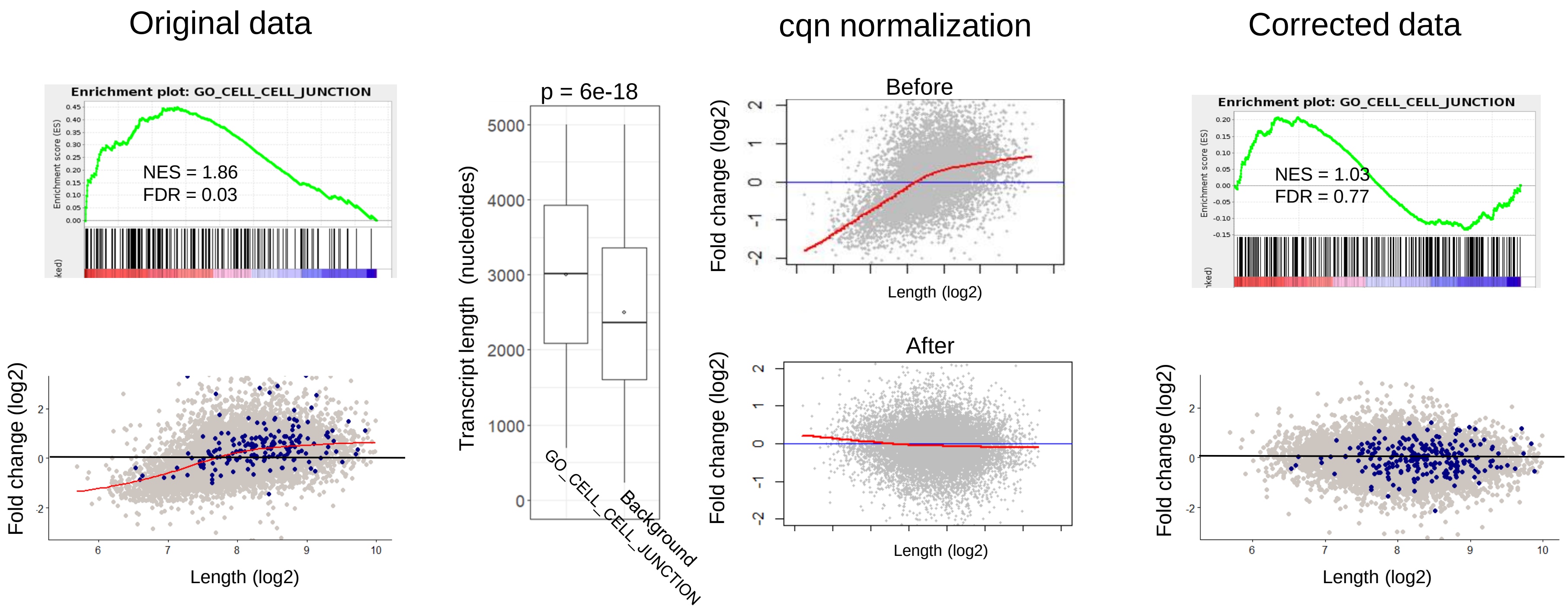

Supplementary Figure 3

j Dataset #8: NCS treated vs. untreated MCF-7 cells (GSE101738): Replicate comparison

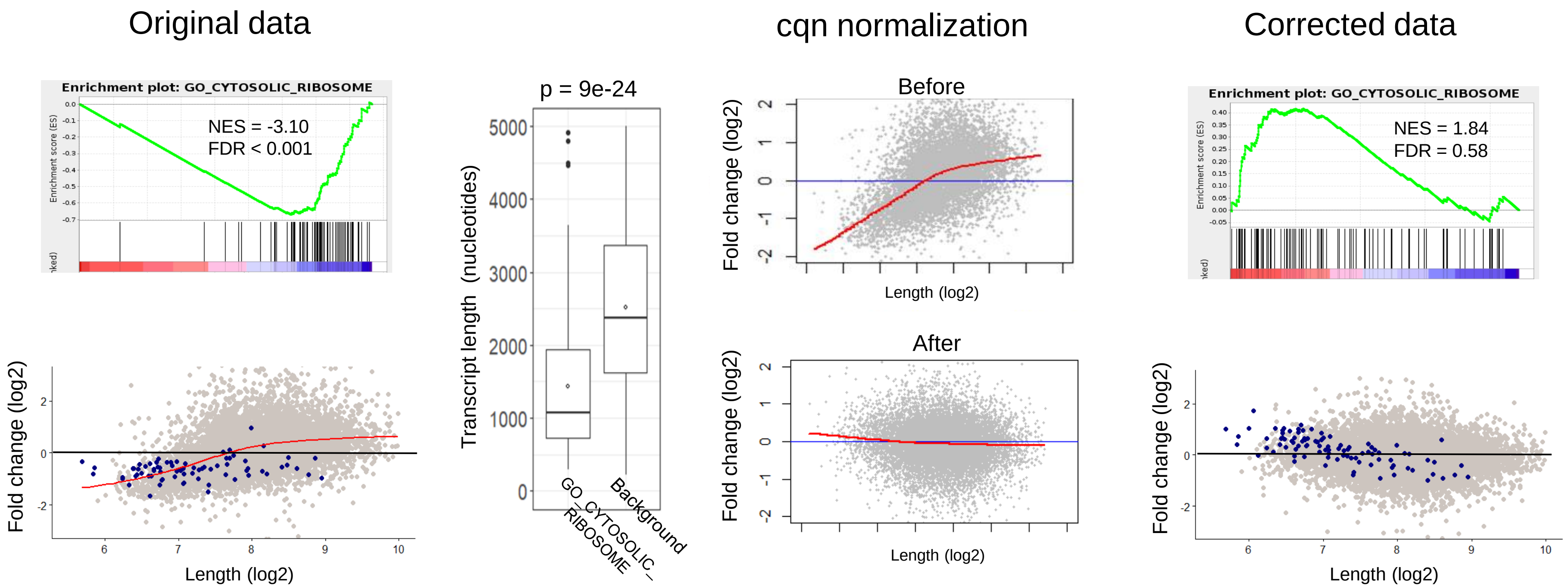

Supplementary Figure 3

k Dataset #8: NCS treated vs. untreated MCF-7 cells (GSE101738): Replicate comparison

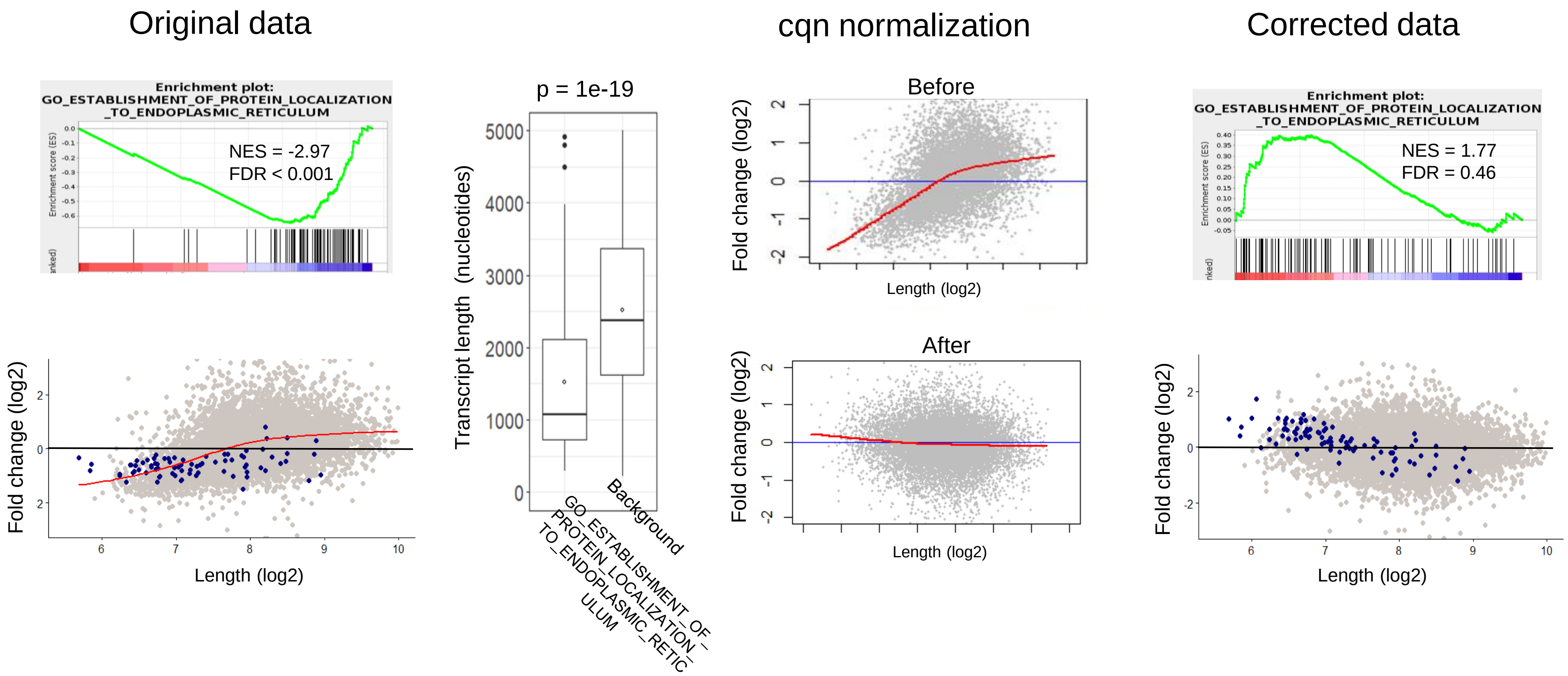

Supplementary Figure 3

I

Dataset #18: AI-10-49 treatment on ME-1 cells (GSE101788): Replicate comparison

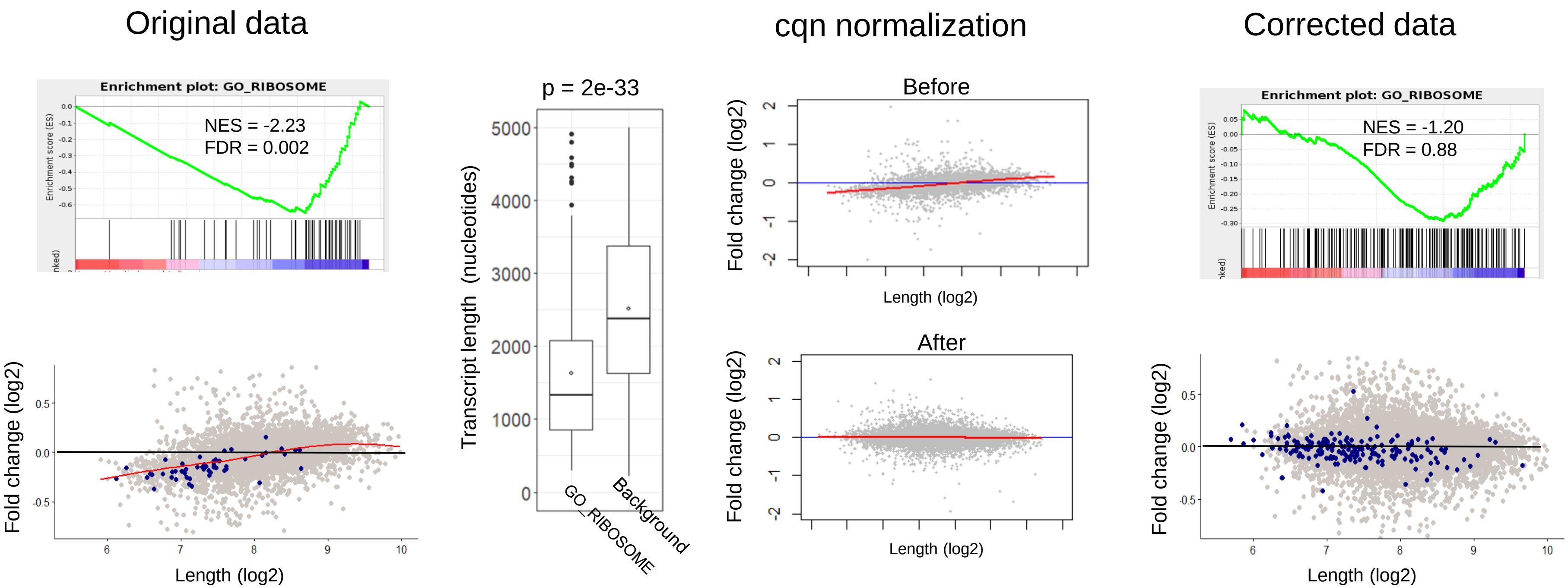

Supplementary Figure 3

m Dataset #18: AI-10-49 treatment on ME-1 cells (GSE101788): Replicate comparison

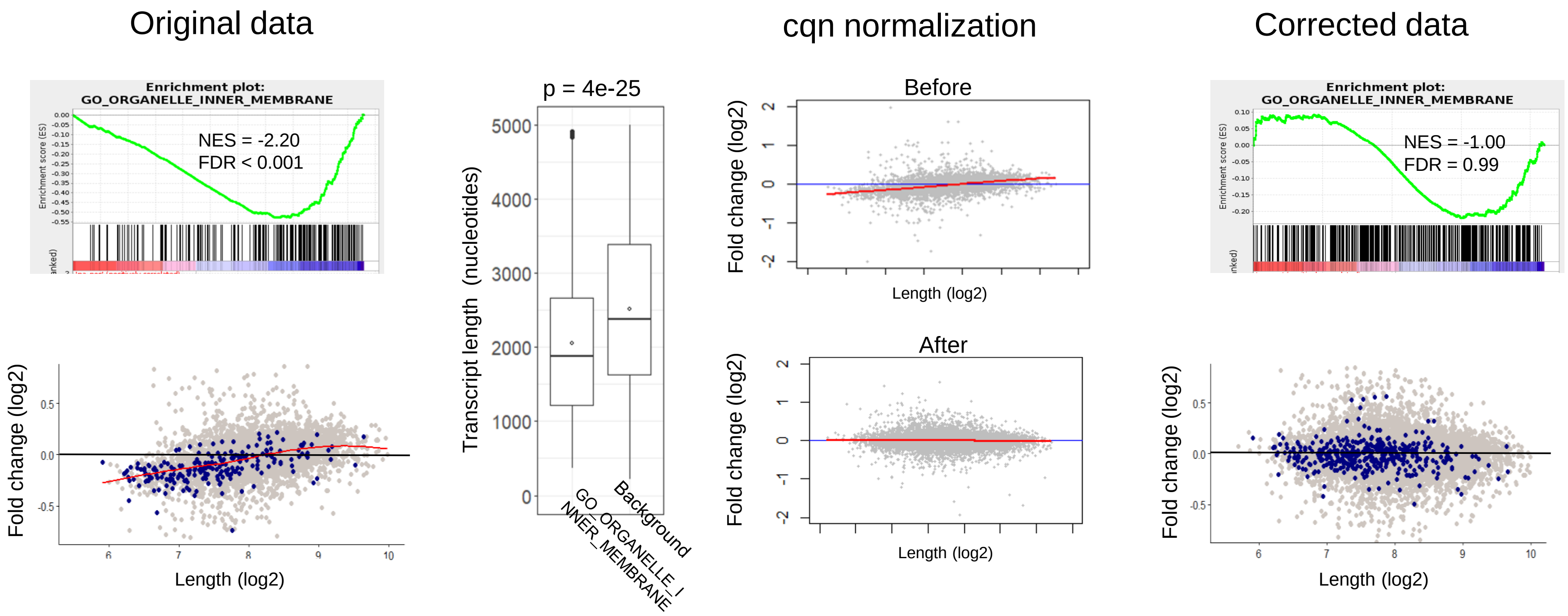

Supplementary Figure 3

n Dataset #18: AI-10-49 treatment on ME-1 cells (GSE101788): Replicate comparison

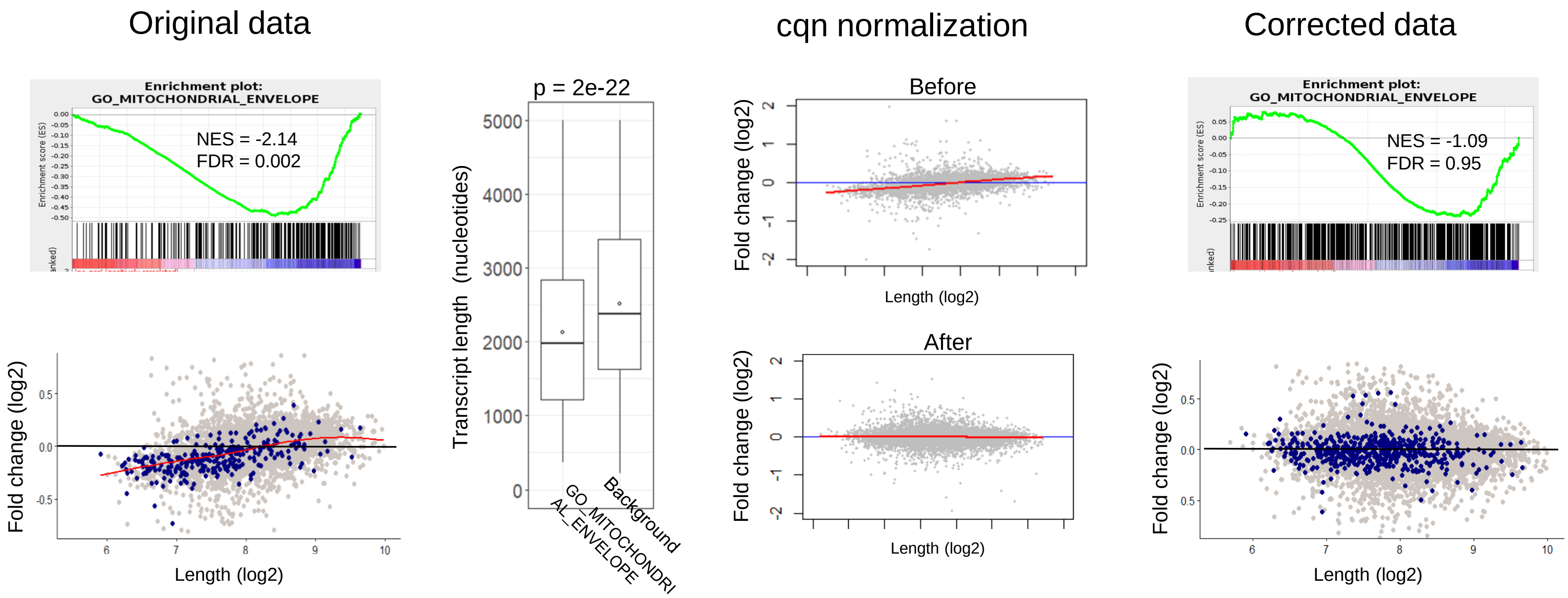

Supplementary Figure 3
