# Supplementary material for: Recurrent functional misinterpretation of RNA-seq data caused by sample-specific gene length bias
Source: PLoS Biol. 2019 Nov 12;17(11):e3000481. doi: 10.1371/journal.pbio.3000481 (PMC6850523; doi:10.1371/journal.pbio.3000481)
Supplement: S4 Fig — (A) (Top left) Sample-specific length bias in the TNFa (GSE64233) dataset. This figure is the same as Fig 1A, showing the comparison between treated and control samples. FC is strongly linked to gene length. (Bottom left) This dataset shows only very minimal sample specific GC bias, indicating that sample-specific length bias is not a mere reflection of GC effects. (Top right) Running cqn with only GC content as sample-specific covariate did not remove the length effect in this dataset. (Bottom right) Adding the sample-specific length covariate to the cqn run completely corrects the length bias. (B) Analysis of the same dataset using EDASeq. (Top) EDASeq run with FQ between-samples normalization and no within-samples normalization shows the absence of GC-content effect (left) and strong length bias (right) in this dataset. (Middle) EDASeq run with within-samples normalization adjusting for GC content followed by FQ between-samples normalization does not remove the length bias. (Bottom) EDASeq run with within-samples normalization adjusting for gene length followed by FQ between-samples normalization effectively corrects the length bias. See S4 Table for results on the 35 RNA-seq datasets. Data underlying the results presented in this figure are provided in https://github.com/ElkonLab/RNA-seq_length_bias/. cqn, conditional quantile normalization; FC, fold change; FQ, full quantile; RNA-seq, RNA sequencing; TNFa, tumor necrosis alpha. (PDF) [file pbio.3000481.s004.pdf]

TNFa dataset (GSE64233)

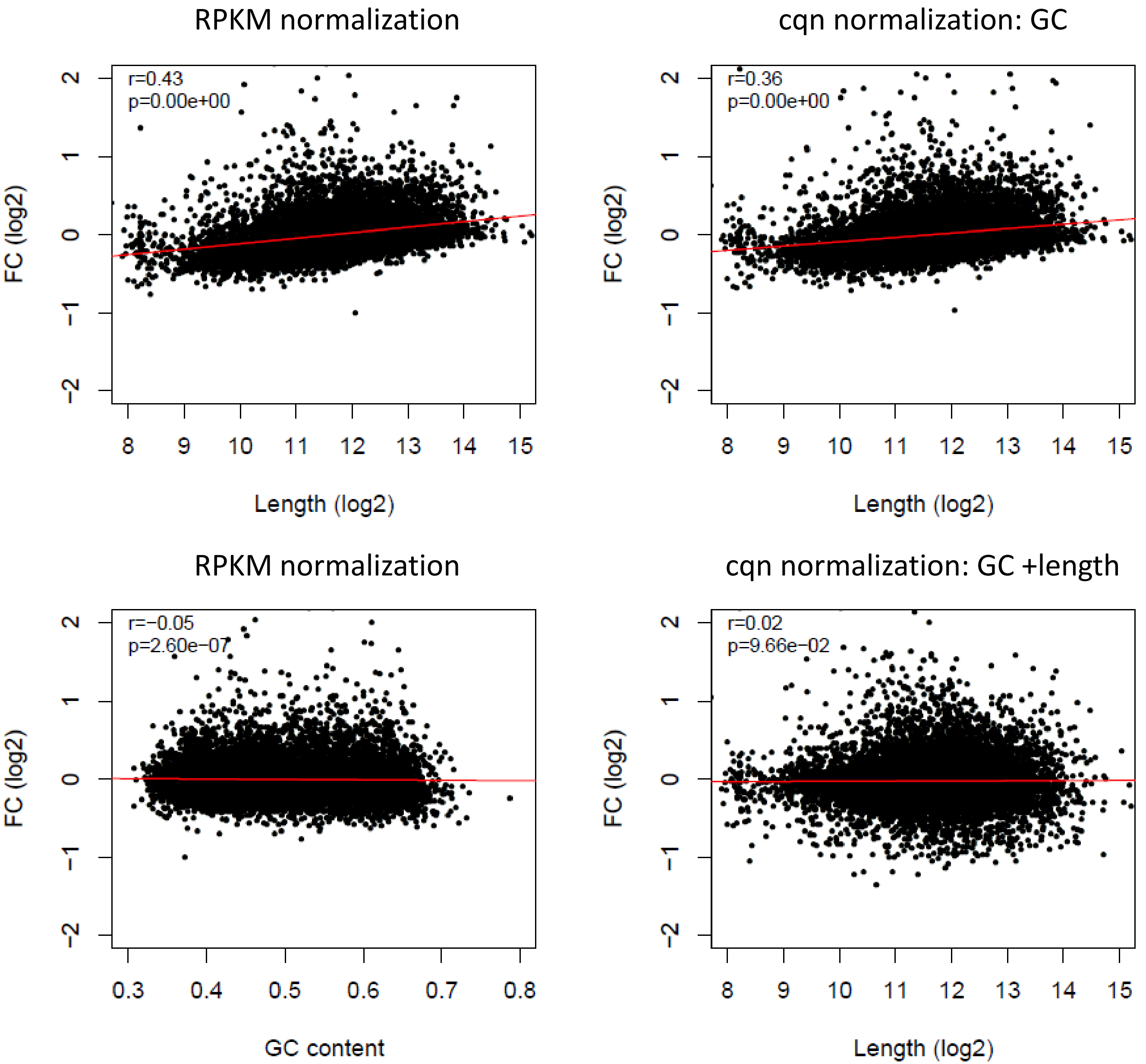

Supplementary Figure 4A

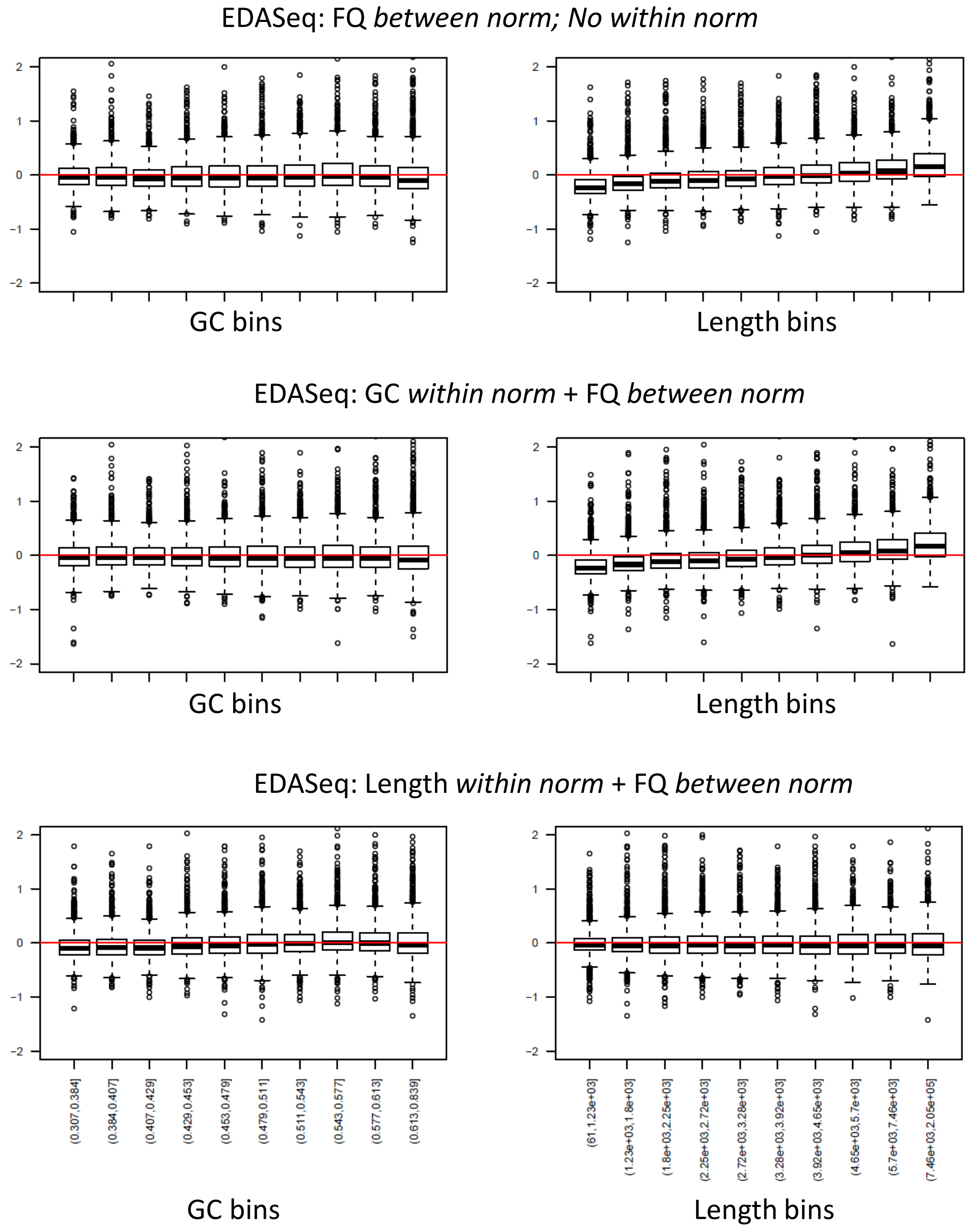

Supplementary Figure 4B
