# Supplementary material for: Recurrent functional misinterpretation of RNA-seq data caused by sample-specific gene length bias
Source: PLoS Biol. 2019 Nov 12;17(11):e3000481. doi: 10.1371/journal.pbio.3000481 (PMC6850523; doi:10.1371/journal.pbio.3000481)
Supplement: S5 Fig — (A-G). Each item shows GSEA results on the original dataset (left) and after the application of cqn (right). The enrichment of the upper gene set is biologically genuine, whereas the enrichment of the bottom gene set is an artifact caused by sample-specific length bias and is canceled by cqn. Data underlying the results presented in this figure are provided in https://github.com/ElkonLab/RNA-seq_length_bias/. cqn, conditional quantile normalization; GSEA, gene-set enrichment analysis. (PDF) [file pbio.3000481.s005.pdf]

a Dataset #20 (GSE76771): Tunicamycin (ER Stress) vs. control

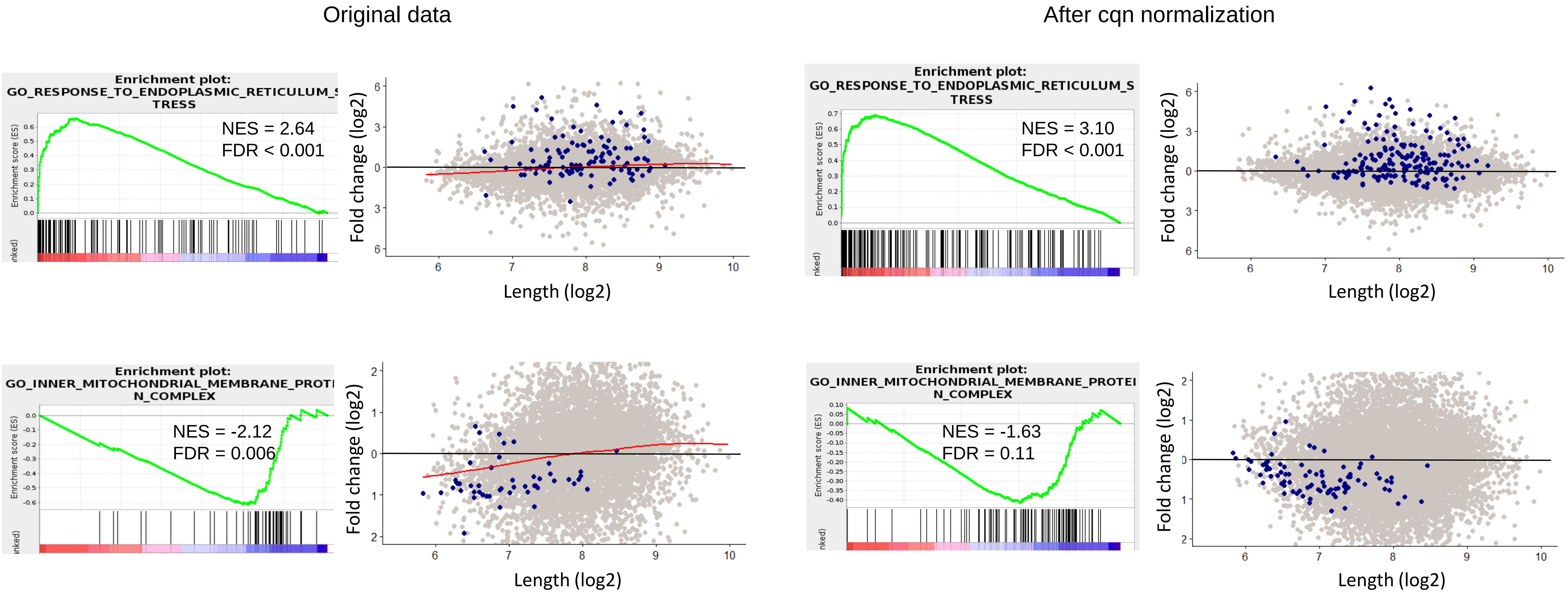

Figure S5

b Dataset #8 (GSE101738): NCS (DNA damage) vs. control

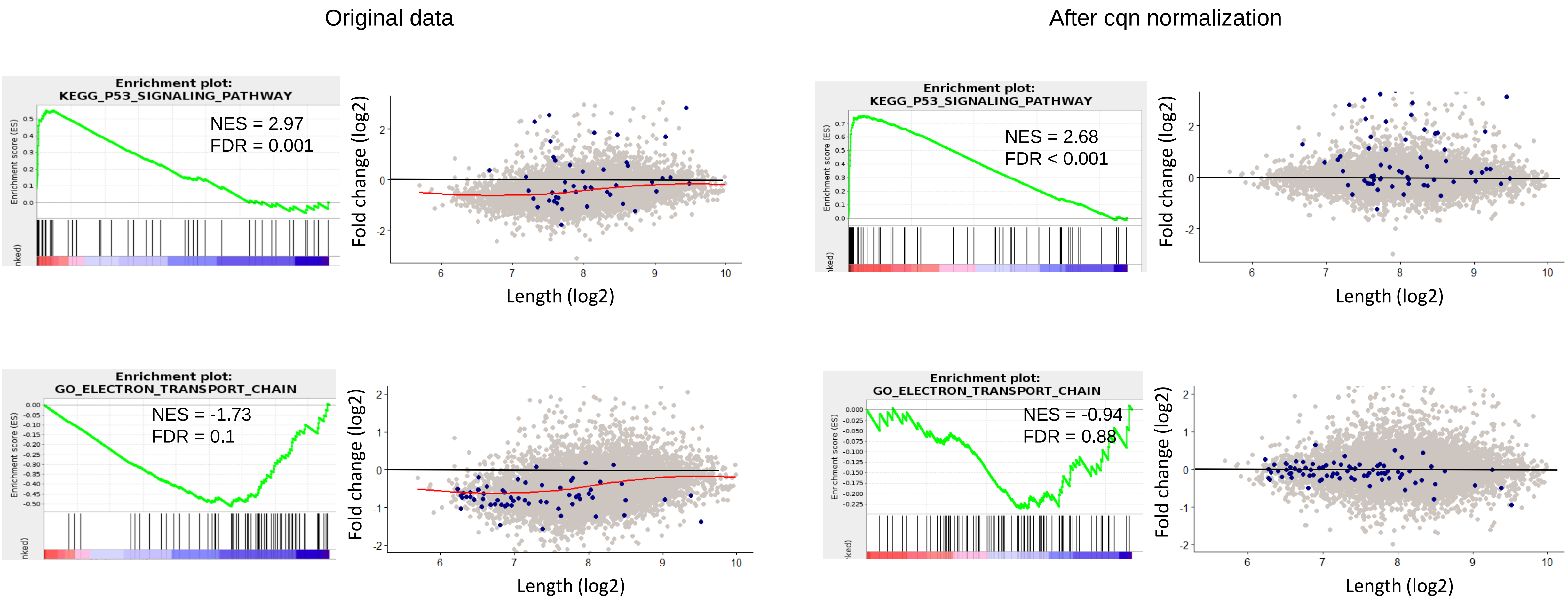

Figure S5

c Dataset #22 (GSE106847): AA147 treatment (ER Stress) vs. control

Original data

After cqn normalization

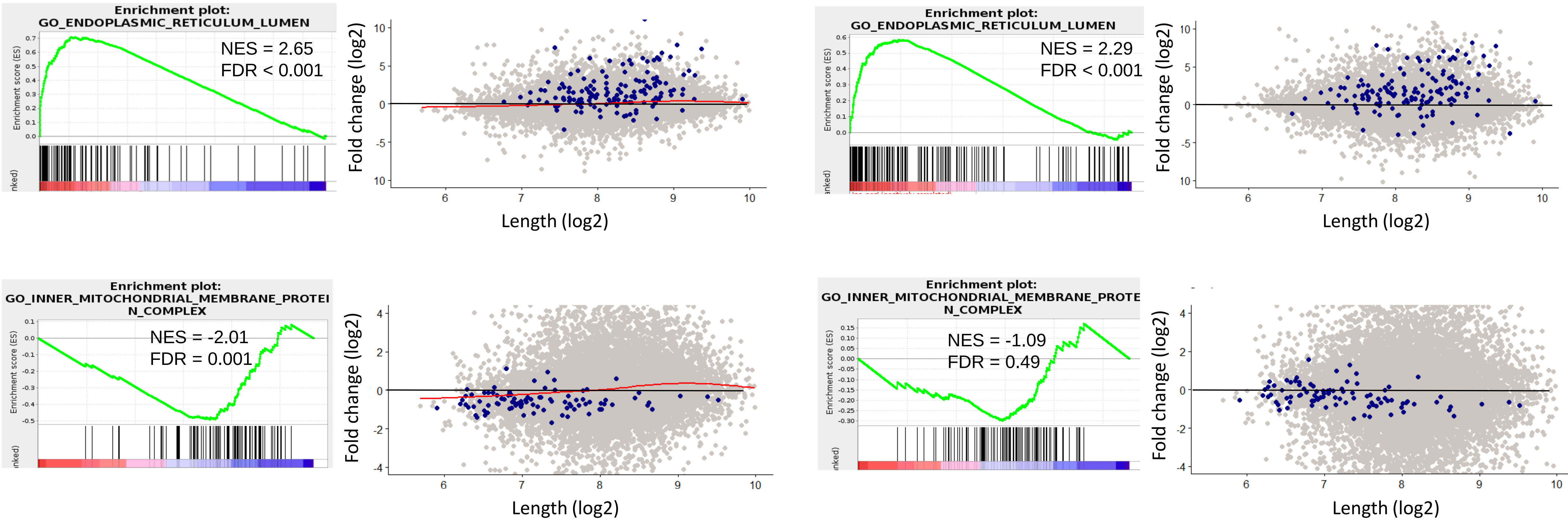

Figure S5

d Dataset #21 (GSE84989): Thapsigargin (ER Stress) vs. control

Original data

After cqn normalization

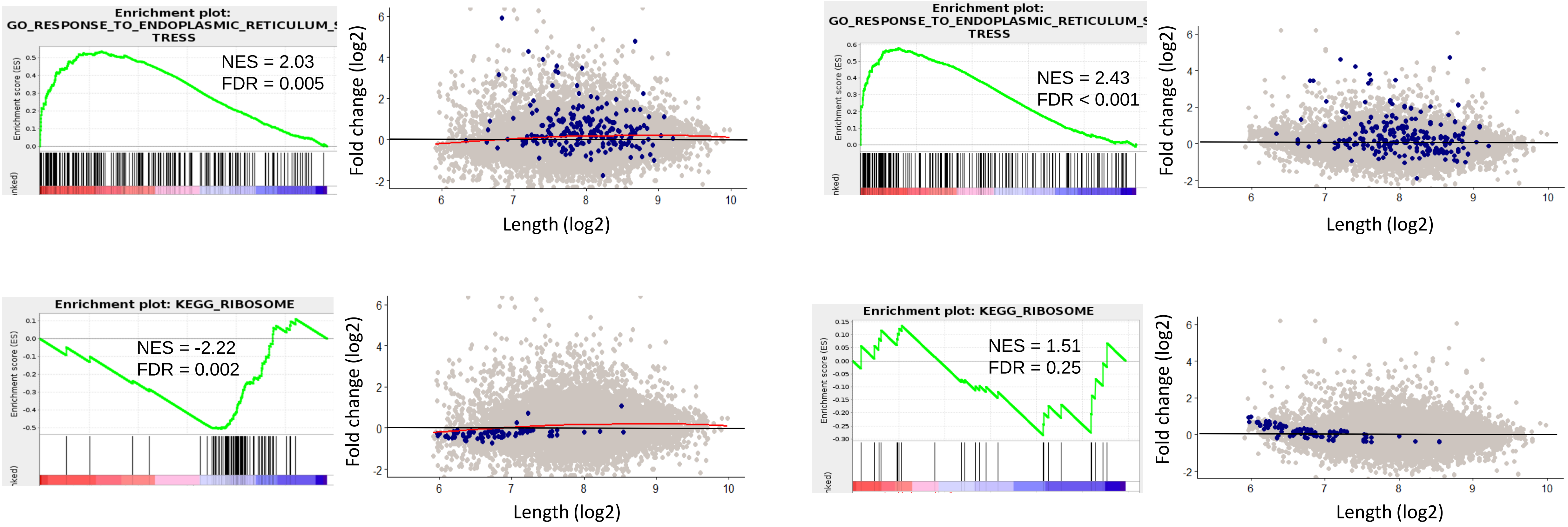

Figure S5

e Dataset #19 (GSE42509): RAS^{G12V} activation (senescence) vs. control

Original data

After cqn normalization

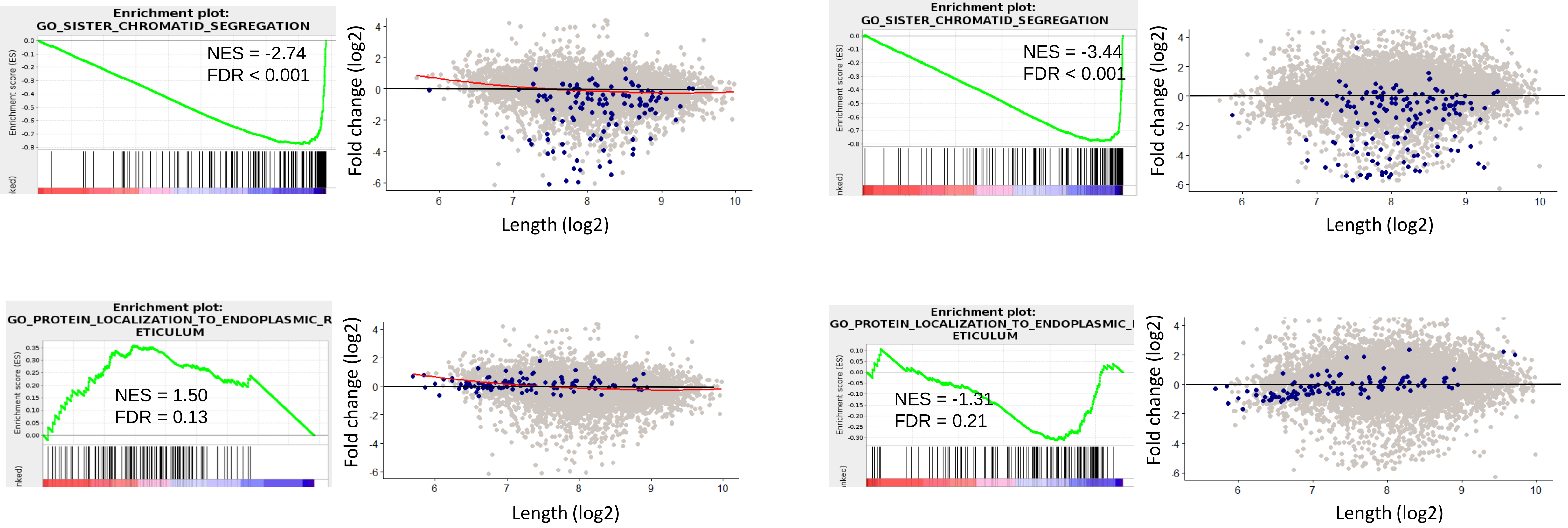

Figure S5

f Dataset #18 (GSE101788): AI-10-49 treatment (apoptosis) vs. control

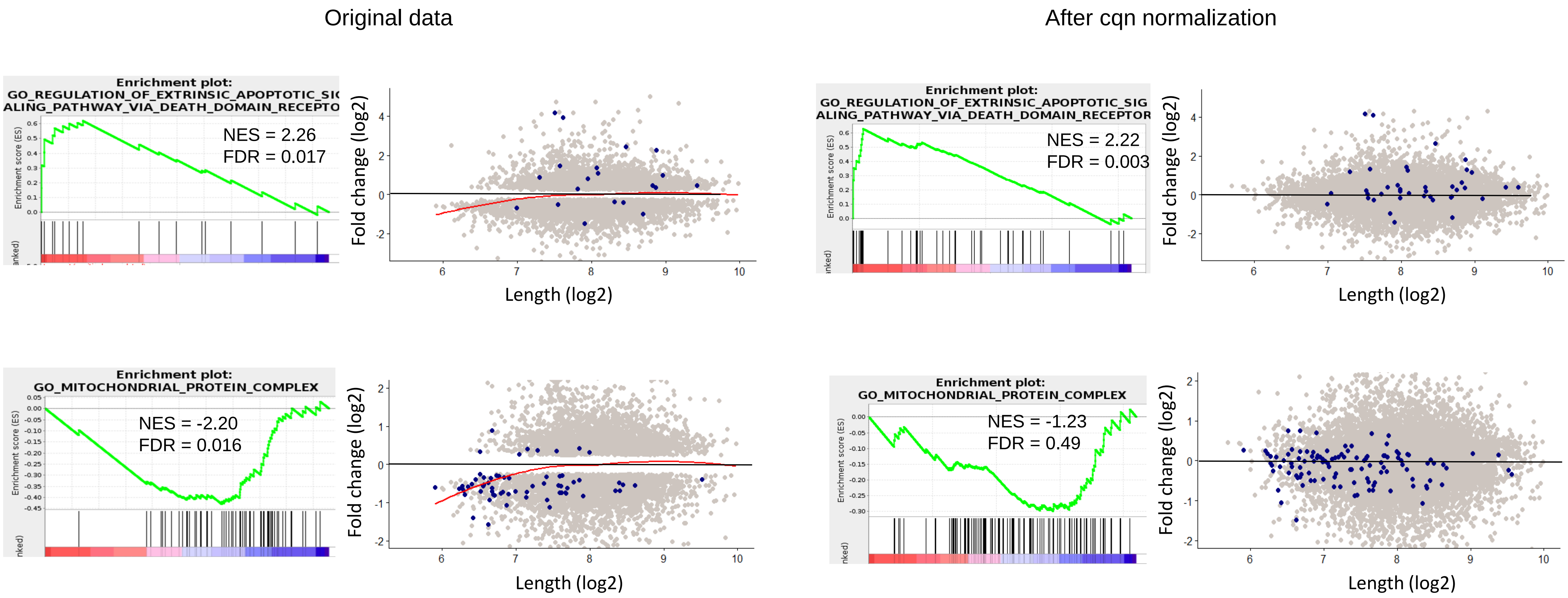

Figure S5
